# Statistical Significance Filtering Overestimates Effects and Impedes Falsification: A Critique of Endsley (2019)

**DOI:** 10.3389/fpsyg.2020.609647

**Published:** 2020-12-22

**Authors:** Jonathan Z. Bakdash, Laura R. Marusich, Jared B. Kenworthy, Elyssa Twedt, Erin G. Zaroukian

**Affiliations:** ^1^United States Army Combat Capabilities Development Command, Army Research Laboratory South at the University of Texas at Dallas, Richardson, TX, United States; ^2^Department of Psychology and Special Education, Texas A&M University–Commerce, Commerce, TX, United States; ^3^United States Army Combat Capabilities Development Command, Army Research Laboratory South at the University of Texas at Arlington, Arlington, TX, United States; ^4^Department of Psychology, University of Texas at Arlington, Arlington, TX, United States; ^5^Department of Psychology, St. Lawrence University, Canton, NY, United States; ^6^United States Army Combat Capabilities Development Command, Army Research Laboratory, Computational and Information Sciences Directorate, Aberdeen, MD, United States

**Keywords:** significance filter, selection bias, p-hacking, meta-analysis, confirmation bias, situation awareness, performance, falsification

## Abstract

Whether in meta-analysis or single experiments, selecting results based on statistical significance leads to overestimated effect sizes, impeding falsification. We critique a quantitative synthesis that used significance to score and select previously published effects for situation awareness-performance associations (Endsley, 2019). How much does selection using statistical significance quantitatively impact results in a meta-analytic context? We evaluate and compare results using significance-filtered effects versus analyses with all effects as-reported. Endsley reported high predictiveness scores and large positive mean correlations but used atypical methods: the hypothesis was used to select papers and effects. Papers were assigned the maximum predictiveness scores if they contained *at-least-one* significant effect, yet most papers reported multiple effects, and the number of non-significant effects did not impact the score. Thus, the predictiveness score was rarely less than the maximum. In addition, only significant effects were included in Endsley’s quantitative synthesis. Filtering excluded half of all reported effects, with guaranteed minimum effect sizes based on sample size. Results for filtered compared to as-reported effects clearly diverged. Compared to the mean of as-reported effects, the filtered mean was overestimated by 56%. Furthermore, 92% (or 222 out of 241) of the as-reported effects were below the mean of filtered effects. We conclude that outcome-dependent selection of effects is circular, predetermining results and running contrary to the purpose of meta-analysis. Instead of using significance to score and filter effects, meta-analyses should follow established research practices.

## Introduction

The goal of meta-analysis is the objective quantitative synthesis of effect sizes from the relevant literature (Mulrow, 1994; Borenstein et al., 2009; Cooper et al., 2009, 2019; Goldacre, 2010; Gurevitch et al., 2018; Corker, 2019). Individual experiments vary in sample size, methodology, measures, and quality, and their results may conflict. Meta-analysis summarizes the magnitude, direction, and variation of effects with potentially greater generalizability than separate studies, and with less bias than the qualitative interpretations in narrative reviews. Glass (2015) recounts that his original motivation for inventing meta-analysis was to provide a more objective alternative to biased narrative reviews in psychology. In particular, Glass was concerned about reviews that used arbitrary criteria such as statistical significance to cherry-pick desired results and exclude undesired results.

In general, selecting results based on statistical significance (i.e., including only results reaching a specified *p*-value) leads to overestimated effect sizes (Lane and Dunlap, 1978; Hedges, 1984; Gelman and Carlin, 2014; Vasishth et al., 2018). Filtering effects using statistical significance^1^ distorts results and impedes falsification (Kriegeskorte et al., 2009; Vul et al., 2009; Ioannidis et al., 2014; Wasserstein and Lazar, 2016; Nelson et al., 2018). Significance filtering in meta-analysis is even more problematic than in single experiments because meta-analysis is used for drawing overarching conclusions across relevant literature. Vosgerau et al. (2019) warn that, “…if a meta-analysis is infused with even a modicum of selective reporting, it becomes an invalid and dangerously misleading tool” (p. 1630).

Here, we critique Endsley’s (2019) use of statistical significance to score and filter “relevant” results from previously published papers in a meta-analytic context. Endsley’s work synthesized multiple aspects (e.g., sensitivity, intrusiveness, and predictiveness for performance) of a cognitive construct called situation awareness (SA; Endsley, 1995a, b, 2015a; Tenney and Pew, 2006). In this critique, we focus on “predictiveness,” which was assessed through SA-performance associations.

Situation awareness can be generally summarized as “knowing what is going on” (Endsley, 1995b, p. 36). More formally, SA is often operationalized with three levels: “… the perception of elements in the environment within a volume of time and space, the comprehension of their meaning, and the projection of their status in the near future” (Endsley, 1995b, p. 36). One widely used theory specifies that SA is probabilistically linked (Endsley, 2000) and even critical to performance (Endsley, 2015a). Thus improving SA is posited to also improve performance (Endsley and Jones, 2011), and SA is often used on its own to assess the effectiveness of different types of training and systems designs (e.g., automation, displays, and interfaces; Endsley, 2019). However, some researchers have raised concerns that SA may be circular and perhaps too vague (Flach, 1995), and that SA’s theoretical relationship to performance may even be unfalsifiable (Dekker and Hollnagel, 2004); for responses see Endsley (2015a, b).

A clear way to quantitatively test these diverging perspectives of SA’s validity, or associations with performance, would be an objective meta-analysis of the relevant empirical literature based on a systematic review. While Endsley (2019) does provide a synthesis of papers reporting SA-performance associations, clear inclusion/exclusion criteria were not specified (see Supplementary Material 1.1), and the analyses were conducted using highly unconventional methods that relied upon significance filtering to score and select the SA-performance associations reported in the literature. Endsley explains the methods as follows:

“Not all SA is relevant to all performance measures. Furthermore, most studies are limited in the number of performance measures assessed, increasing the likelihood that some SA metrics may not have the relevant performance metrics for comparison. Therefore, *this meta-analysis assesses whether any SA measure was predictive of any performance measure in each study* [emphasis added].” (p. 7)

These methods produced the following two metrics:

(1) Predictiveness score. This was an overall score assigned to each paper based on the reporting of *at-least-one* significant (or marginal) effect reported in the paper using one-tailed *p*-values for positive correlations:
  (a) Score of +1: There was at least one significant effect (*p* < 0.05; directional *r* > 0) reported in the paper.
  (b) Score of +0.5: There were *no* significant effects, but there was at least one marginally significant effect (*p* < 0.10; directional *r* > 0) reported.
  (c) Score of 0: Only assigned when *all* reported effects in the paper were non-significant (*p* ≥ 0.10; directional null for non-positive effects *r* ≤ 0).
  The predictiveness score represents an unorthodox form of a vote-counting procedure, which even in its standard form is no longer recommended. It is particularly problematic here because the majority of included papers reported *multiple effects*. Traditional or typical vote-counting is for a single effect size per paper: each predicted significant effect receives +1, each non-significant effect receives 0, and each significant effect opposite to the prediction receives −1 (e.g., Bushman and Wang, 2009). In contrast, Endsley (2019) uses an atypical *at-least-one* criteria vote-counting method^2^. For example, a paper reporting 10 effects would receive the same score of +1 if all 10 out of 10 effects were significant, or if only 1 out of the 10 was significant. Furthermore, the choice to use *p*-values to score papers is perplexing because actual effect sizes (not just significance) were reported for SA-performance associations in nearly all included papers.
(2) Aggregated filtered effect size: This was an overall effect size calculated for each paper using the simple average of only the significant and marginally significant effects within that paper. Even when the paper reported non-significant effect sizes in detail, they were filtered out and thus not included in this average.

As described in the quote above, the primary justification provided for filtering effects was “Not all SA is relevant to all performance measures” (Endsley, 2019, p. 7). The idea that a deterministic relationship cannot be expected for probabilistic phenomena is reasonable; however, “relevance,” here was outcome-dependent because it was determined entirely by statistical significance^3^ This is an example of confirmation bias in statistics, or only “looking for evidence consistent with theory” (Bishop, 2020b, p. 4). Circular logic, using a specific hypothesis for outcome dependent selection of effects, makes falsification nearly impossible. For example, one might take the opposite stance that only non-significant effects were “relevant,” perhaps providing the (factually correct, but flawed) justification that the majority of reported effects were non-significant. In this case all directional significant effects would be incorrectly excluded.

The methodological issues with selection bias in Endsley (2019) are concerning and raise the question: How much does it matter? In other words, how much does selection based on statistical significance quantitatively impact results in a meta-analytic context? In this critique, we evaluate and compare results using significance-filtered effects versus analyses with all effects as-reported. First, we describe our dataset of previously published papers included in Endsley (2019) and our inclusion/exclusion criteria. Second, we use simulations to demonstrate non-trivial predictiveness scores with a medium effect size and even with a true effect size of zero. Third, we illustrate that selection of effects using (marginal) significance imposes deterministic boundaries: guaranteed minimum values for effect sizes bounded by sample size. Fourth, we compare significance-filtered means to meta-analytic means using all reported effects, regardless of statistical significance. Fifth, we evaluate the proportion of all effects below the significance filtered means. Last, we provide an overall discussion and recommendations.

## Methods and Results

### Dataset

The purpose of this paper is to directly compare significance filtering to inclusion of all reported effects, which involves re-examining the papers considered by Endsley (2019), rather than conducting a systematic review of the literature. The following minimal criteria were used for paper inclusion (for details on excluded papers see Supplementary Material 1.1):

(1) The paper was one of the 46 previously published papers included in Endsley, Appendix C: Predictiveness of SA Metrics. Note that papers were eligible for inclusion even if they were assigned a predictiveness score of “—” or “0” by Endsley because our inclusion criteria were not dependent on statistical significance.
(2) The paper reports association(s) between SA and task performance (e.g., decision accuracy) as a correlation or an effect size that could be converted to a correlation. Seven of the 46 papers did not meet this inclusion criterion.
(3) Sufficient data: The paper was not unique in its use of an SA measure. In other words, the specific SA measure assessed in the included paper was also used in at least one other paper. This criterion completely excluded the single paper to assess SALSA (originally in German, translated into English as “Measuring Situation Awareness of Area Controllers within the Context of Automation” Hauss and Eyferth, 2003, p. 442), and partially excluded the single paper that assessed “real-time probe,” but its results for other measures were retained. See Endsley for details about all measures.

The above criteria illustrate why it was not possible for us to here analyze all of the same papers analyzed in Endsley (2019) in a way that provided meaningful results.

Using the above criteria, we included 38 papers out of the 46 unique papers in Endsley, Appendix C, excluding 8 papers completely and 1 partially (see Supplementary Material 1.1 for details). Throughout this paper, we draw comparisons between analyses using all reported effects from these 38 papers and what we refer to as the *filtered means* from Endsley’s analysis; shown in Table 1^4^.

**TABLE 1:** Results for aggregated effect sizes for each SA measure as reported in Table 5, p. 13, Endsley (2019) and our calculations of overall effects using that data.

Method (SA measure)	Mean pearson’s *r*	Confidence interval
End of trial	*0.533*	*[0.522, 0.545]*
SAGAT	*0.459*	*[0.432, 0.487]*
SPAM	*0.411*	*[0.368, 0.454]*
Overall simple average (our calculation): Filtered overall mean	0.46	

*SAGAT, Situation Awareness Global Assessment Technique, SPAM, Situation Present Assessment Method. We calculated the filtered overall mean using a simple average of z-transformed values in Appendix C, Endsley, excluding the previously mentioned two measures assessed only once. No confidence interval was calculated for the filtered overall mean because we were unable to reproduce the confidence intervals in Endsley, Table 5 using data from Endsley, Appendix C (see Supplementary Material 1.8).*

To enable more direct comparison with Endsley (2019), here we included 12 papers in our set of 38 papers that would be unlikely to meet inclusion in a systematic review: See Supplementary Material 1.2 for details. Ten of these published papers (with 79 total effects) reported statistical results from repeated measures data that were incorrectly modeled as independent observations/participants. We contend such overfit results are generally uninterpretable. Overfit results tend to yield specious results for effect sizes and their corresponding *p*-values, typically underestimating standard error/confidence intervals (Babyak, 2004; Aarts et al., 2014). Moreover, it is also possible to have overestimated, instead of underestimated, variance around parameters (Kenny and Judd, 1986), and even different point-estimated effect sizes for regression on data that are averaged versus overfit: See Figure 6 in Bakdash and Marusich (2017) for a visualization of overfitting. In addition, three papers (one paper also had overfit results) assessed SA and performance at the team-level. Stanton et al. (2017) posited that theories for individual and team SA have similarities and differences, thus they are not necessarily interchangeable (also see Supplementary Material 1.2).

**FIGURE 1:**
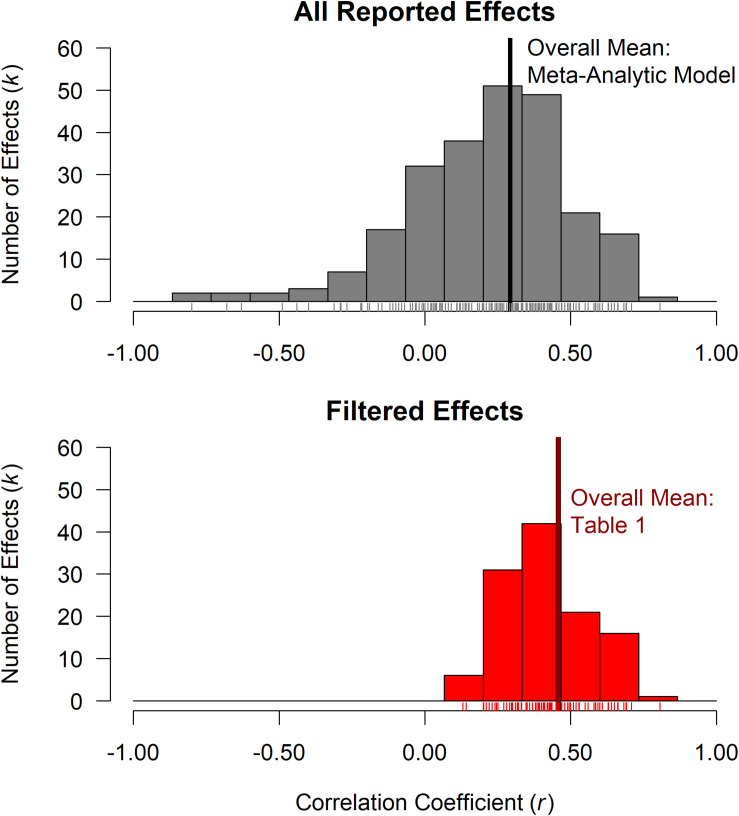
**Top Panel:** Distribution of the 241 effects from the 38 papers included in our dataset. Values of individual effect sizes are indicated by ticks above the *x*-axis with a thick vertical line for overall mean estimated using a meta-analytic model. **Bottom panel:** Distribution of significance filtered effects from the above dataset, the overall mean from Table 1 (*r* = 0.46). Note the restriction of range with significance filtering.

In our 38-paper dataset, we used all 241 effects as they were reported in the papers, regardless of statistical significance or overfitting (see Supplementary Material 1.2). It is important to note that in this work we only included non-significant effects that were reported in detail in the papers (e.g., we did not include any results generically described as *p* ≥ 0.05 or patterns of selective omission), see section “Limitations” for more information. The top panel of Figure 1 shows all 241 reported effects, and the bottom panel shows significance-filtered effects – the 117 filtered effects that met one-tailed significance for *r* > 0.00. Note the limited range of filtered effects, with an overall mean (*r* = 0.46)^5^ approaching a large positive effect size. While the filtered effects and their means are empirically derived, selection using significance discards 51.45% (124 out of 241) of all reported effects from our 38 paper dataset.

**FIGURE 2:**
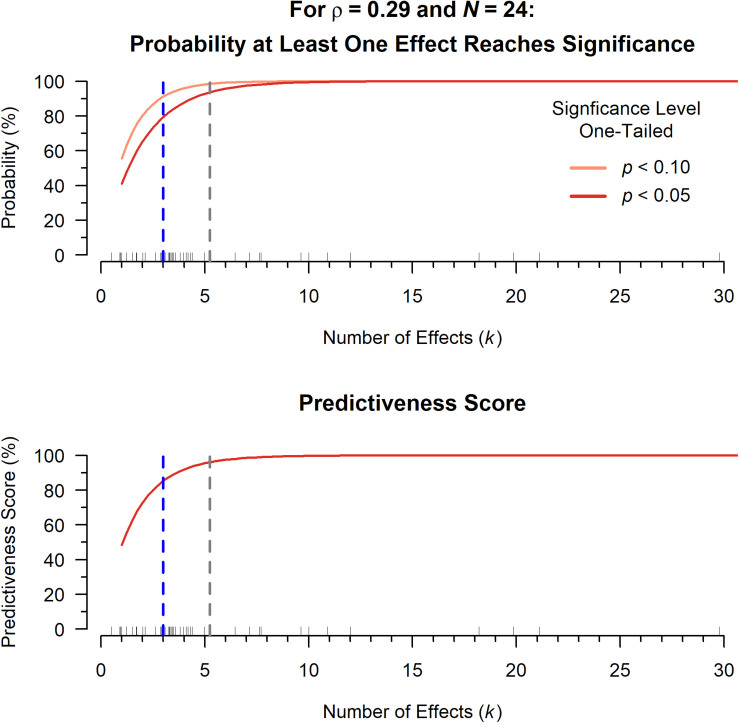
**Top:** The probability that at least one effect in a given paper is significant or marginally significant as a function of the number of effects per paper (*k*), defined by 1−(1−δ)^*k*^, where power δ is determined using the significance level (α = 0.05, dark red, or α = 0.10, pink), the empirical sample size (*N* = 24), and the empirical effect size (ρ = 0.29). The median (blue) and mean (gray) number of effects per paper are shown as dotted lines. Individual *k* values for the papers in our data set are indicated by ticks above the *x*-axis. **Bottom:** The expected value of the predictiveness score as a function of *k*, again using the empirical sample size (*N* = 24) and effect size (ρ = 0.29).

In comparison to the nearly large effect for the overall filtered mean, the overall effect size from a meta-analytic model, using all 241 reported effects and taking into account the dependencies of multiple effects from each paper (described in section “Comparison of Significance Filtered Means Versus Means of As-Reported Effects”), was considerably less at *r* = 0.29 (a medium effect size for a correlation coefficient). The 38 papers in our dataset had a median reported sample size of *N* = 24, see Supplementary Material 1.4, Figure 1. Median values for the number of effects per SA measure per paper was *k* = 3, see Supplementary Material 1.4, Figure 2.

#### Data Cleaning and Analyses

We performed data cleaning and analyses using the statistical programming language *R* (R Core Team, 2020). Data cleaning was primarily conducted using the *tidyverse* package (Wickham et al., 2019) and effect sizes were converted using *esc* (Lüdecke, 2019). To calculate significance for different effects across various sample sizes, we used the *pwr* package (Champely, 2020). We primarily fit meta-analytic models using *metafor* (Viechtbauer, 2010). In addition, we used *club Sandwich* (Pustejovsky, 2020), and *robumeta* (Fisher and Tipton, 2015; Fisher et al., 2017). We modified the *ggforestplot* package to create Figure 4 (Scheinin et al., 2020). The proportion of effects above and below filtered means were estimated using the *MetaUtility* package (Mathur et al., 2019) and the *boot* package (Canty and Ripley, 2020). For a list of all R packages, see Supplementary Material 1.5.

This work uses a subset of data and code for analyses from Bakdash et al. (2020a, b), a preprint of a meta-analysis of SA-performance (Bakdash et al., 2020c). Data from the papers included here were checked by multiple coders, see Bakdash et al. (2020c) for details. Our results are reproducible using our materials on the Open Science Framework (Bakdash et al., 2020d) or with the Code Ocean platform (Clyburne-Sherin et al., 2019) using our capsule (Bakdash et al., 2020e).

### Predictiveness Score and Vote-Counting

As described earlier, the predictiveness score implemented in Endsley (2019) was a single value of either 0, +0.5, or +1 assigned to each SA measure assessed in a paper (e.g., SPAM or SAGAT), based on the presence of at least one significant (Score of +1) or marginal (Score of +0.5) effect. This was one-tailed, for positive effects only. A score of 0 could be assigned if and only if *no effects* reported in the paper for a given SA measure reached marginal significance. A paper reporting only non-significant effects would be extraordinarily rare given publication bias and the type I error rate with multiple uncontrolled comparisons, see section “Predictiveness Score: Type I Error.” In this section we review the reasons that even standard vote-counting procedures are now widely discouraged, and subsequently describe the concerning statistical implications of this unorthodox method of vote-counting.

#### Traditional Vote-Counting

The predictiveness score implemented by Endsley (2019) represents an atypical version of a vote-counting procedure. Vote-counting has traditionally been used in cases where there is a single effect size per paper: each predicted significant effect receives +1, each non-significant effect receives 0, and each significant effect opposite to the prediction receives −1 (e.g., Bushman and Wang, 2009). The votes for each paper are then summed to create an overall score with the category containing the majority of votes deemed the “winner.”

Even the traditional form of vote-counting, for a single effect per paper, is now considered an antiquated methodology (Mathur and VanderWeele, 2019). Borenstein et al. (2009) declare that there is no reason to ever use it, especially when the information is available from primary-level studies that could be used to calculate meta-analytic effect sizes. Vote-counting is problematic for a variety of reasons. First, it does not consider sample sizes when yielding a vote; a small study with a statistically significant result gets the same consideration as a large study that fails to reach traditional significance levels. Further, vote-counting does not quantify the magnitude of difference between a result that wins a vote and one that fails to do so; a study with an obtained *p*-value of 0.051 receives a 0 vote, whereas a study with an obtained *p*-value of 0.049 receives a +1. Adding a vote of +0.5 for marginal significance does not address this problem, rather it creates an additional arbitrary category. Vote-counting uses arbitrary cut-offs with *p*-values, thus it largely ignores uncertainty in parameter estimates as well as the distinction between statistical (*p*-values) and practical (effect sizes) significance (Borenstein et al., 2009; Gurevitch et al., 2018).

Statistical power is another major concern with traditional vote-counting; it is generally underpowered, assuming there is selection bias in the reported results. As Friedman (2001) noted, “… a vote-count review is likely to yield the wrong conclusion if most studies in a particular area of research have power less than 0.5” (p. 161). Hedges and Olkin (1980) note yet another problem with traditional vote-counting: counterintuitively, statistical power decreases as the number of analyzed results increases.

For these reasons, traditional vote-counting and other quasi-quantitative methods have poor validity. Widely used meta-analytic guidelines caution against the use of quasi-quantitative methods (PRISMA-P Group, Moher et al., 2015). Similarly, the Cochrane handbook (Higgins et al., 2019), considered a gold standard for meta-analysis in health science research, calls vote-counting an “unacceptable synthesis method” (p. 329).

We show that Endsley’s (2019) variation of vote-counting is even more problematic than traditional vote-counting because the *at-least-one* method selects only the results deemed predictive using directional significance. In the next section, we use simulations for the predictiveness score to demonstrate how as the number of effects per paper increases the score can only rise. This shows that with multiple effects, the predictiveness score will be near perfect even with a medium effect size.

#### Predictiveness Score: Type I Error

Despite the well-known issues with traditional vote-counting methods described above, it may be difficult to intuit the implications of using this scoring method to synthesize papers with multiple effects. Here we simulate the expected probability of obtaining a predictiveness score of +1 or +0.5, given the median sample size and number of effects included in this dataset. We perform this simulation using a population effect size of ρ = 0.29: The overall effect obtained from a multilevel meta-analytic model using all effects as-reported from the 38 papers included here (see section “Comparison of Significance Filtered Means versus Means of As-Reported Effects” for more details).

When there is only a single effect in a paper, the probability of obtaining a statistically significant effect is a straightforward power calculation^6^. Assuming a true effect size of ρ = 0.29 and a median sample size^7^ of *N* = 24, the calculated power (probability of finding a one-tailed significant effect, *p* < 0.05) for a single effect is 41.02%. This is to be expected with a medium effect size.

However, most included papers reported multiple effects (median of *k* = 3). If three correlations were performed in a given paper, there are eight possible outcomes: all are significant, only the first is significant, only the second is significant, only the third is significant, only the first and second are significant, only the first and third are significant, only the second and third are significant, or none are significant. The probability of finding at least one significant effect (a predictiveness score of 1 would be equivalent to the probability that the eighth outcome does not happen) is quite high:

(1) Probability of at least one significant effect: 1−(1−41.02%)^3^ = 79.48%
(2) Probability of *no* significant effects: (1−41.02%)^3^ = 20.52%

This may look familiar to some readers; the probability is the same as the Type I error rate (also, called the familywise error rate; both assume the null is true). The formula for Type I error (Cohen et al., 2003), the probability of finding at least one significant effect, is: 1−(1−δ)^*k*^, where δ is statistical power^8^ and *k* is the number of multiple comparisons (i.e., the number of effects per paper here). The *at-least-one* predictiveness score is simply a weighted version of Type I error, see equations in Supplementary Material 1.6.

Figure 2 (top) shows this relationship between *k* and the probability of finding at least one significant effect (as well as the same relationship for marginal effects), still assuming ρ = 0.29 and *N* = 24. The median and mean number of effects for papers in the current dataset are highlighted. Note that the probability to detect at least one significant effect grows quickly: 41% to about 80% with the number of effects increasing from 1 to 3. With more effects in a given paper, the probability of finding one instance of significance quickly approaches 100%. In terms of the predictiveness score, increasing the number of effects while using the *at-least-one* method can only increase the probability of assigning a score of +1. Figure 2 (bottom) shows the expected value of the predictiveness score, which is calculated by multiplying vote values by their corresponding probabilities. Because the predictiveness score uses probabilities as weights, the expected value for the predictiveness score also rises sharply as the number of effects increases.

**FIGURE 3:**
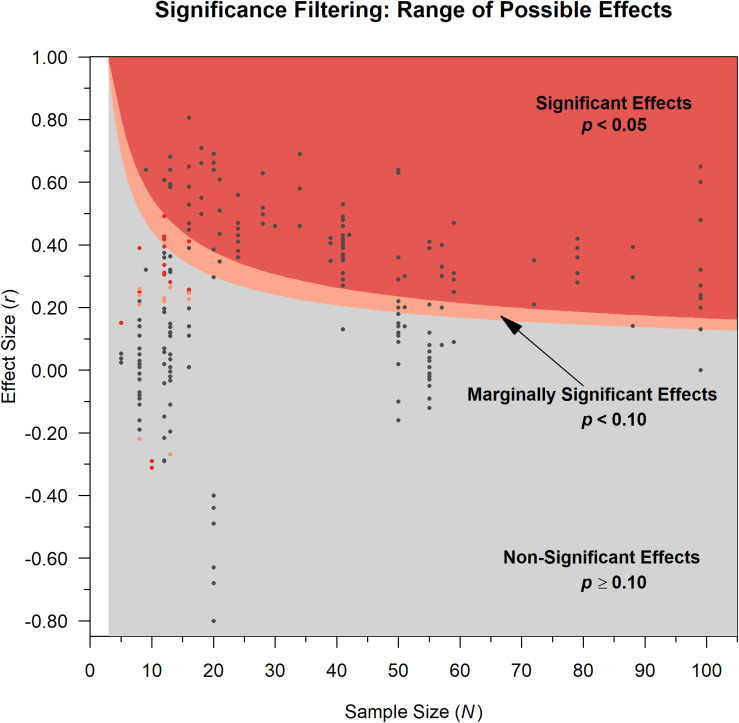
For a one-tailed, positive correlation: The shaded areas are the possible range of effect sizes for significant (dark red), marginally significant (pink), and non-significant results from papers using the sample size reported in each paper/dataset. The lowest values of the red and pink shaded areas depict the guaranteed minimum effect sizes. Dark gray dots show the actual effect sizes (*y*-axis) and corresponding sample sizes (*x*-axis) as-reported for results of the individual papers in our dataset. Red and pink dots indicate overfit results (excessive degrees of freedom, see section “Dataset” and Supplementary Material 1.2), that only reached two-tailed significance or marginal significance, respectively, due to overfitting. For example, one paper has a stated sample size of 10 participants but reports a result of *r*(52) = 0.32, *p* < 0.02; a Pearson correlation has *N* – 2 degrees of freedom so this should be *r*(8), see Supplementary Material 1.2 for more information. Note with one-tailed tests, all non-positive effects will be non-significant and thus filtered out based on Endsley’s described method. One paper with *N* = 171 is not shown.

The concerning aspects of the relationship between number of effects and predictiveness score are perhaps made clearer by assuming a true effect size less than or equal to 0 (see Supplementary Material 1.6, Figure 3). Even in this boundary condition, the predictiveness score will always monotonically increase as a function of *k*, indicating that the predictiveness score has no statistical value.

### Guaranteed Minimum Effect Sizes

Significance filtering can be conceptualized as fishing, dredging, or cherry picking by skimming the “desirable” effects off the top. This provides guaranteed minimum effect sizes because it discards “undesirable” non-significant effects below. Thus, the aggregated effect size from each paper will always be at or above a certain minimum depending on the sample size used in the paper. Including marginal significance (*p* < 0.10), in addition to significance (*p* < 0.05), may seem to provide more precision than dichotomizing (significance versus non-significance). However, the difference is minute. The marginal significance filter simply produces slightly lower bounds on the minimum effect sizes.

According to Endsley’s (2019) methodology, any papers that report no significant effects are discarded from the aggregation of effect sizes because such results are not “relevant.” That is, only papers with at least one significant or marginal effect size meet inclusion. Further, to calculate the average effect size for each paper, only the marginal and significant effects are included. All non-significant effects, even those reported in detail, were not included in the average. This artificially truncates the variability in effect sizes and effectively means that the “average” effect size for each paper in Endsley’s analysis can be no lower than the threshold for significance (often called the “critical” effect size value). This threshold is determined by the combination of the alpha level and sample size. Any reported non-significant effect sizes that are lower than these values were simply filtered out.

Figure 3 shows the range of possible effects that can be obtained from a paper, given the one-tailed significance filtering described in Endsley’s methods. If non-significant results (the gray region) are filtered out of analysis, the only possible values are those in the colored regions, where the red region shows the possible effects that meet significance (*p* < 0.05) and the narrow pink area represents effects that only reach marginal significance (*p* < 0.10). As sample size increases, the guaranteed minimum effect size decreases. However, in this dataset, sample sizes tended to be limited; the median was *N* = 24.

Researchers routinely consider Type I error (a false positive) and Type II error (a false negative) when developing hypotheses and conducting power analyses to set an alpha level and sample size. However, because of the rarity of no true effect for non-directional tests in social science research (Cohen, 1994), an alternative conceptualization of errors focuses on estimation of effect sizes: Type S (sign) error and Type M (magnitude) error (Gelman and Carlin, 2014). A Type S error is the probability that the direction of an estimated effect is inaccurate. For example, researchers find a positive relationship between variables X and Y but the two variables are in fact negatively related. A Type M error is the extent to which an estimated effect size is overestimated: the exaggeration ratio. For example, researchers estimate a large effect (*r* = 0.60) but the true effect size is small (*r* = 0.20), resulting in a Type M error of three. Type S and M errors are common in small samples, which tend to produce unstable, widely varying effects (Button et al., 2013; Schönbrodt and Perugini, 2013; Gelman and Carlin, 2014; Loken and Gelman, 2017).

At a general level, misestimation (typically overestimation) of effects can also occur due to publication bias, which is known as the file drawer problem (Rosenthal, 1979). Researchers, reviewers, and journals favor publishing papers with mostly or even all significant (i.e., typically *p* < 0.05) results, and rejecting papers with non-significant results; hence, many published effect sizes likely reflect overestimates (Kühberger et al., 2014; Luke, 2019). Publication bias is also a form of significance filtering, obscuring judgments on the practical significance of an effect and often hindering attempts to replicate and extend previous work if published effect sizes do not reflect true effect sizes. The methods employed in Endsley (2019) have a similar effect as publication bias, except the filtering is universally applied to each reported result in every included paper. Therefore, a Type M error is essentially guaranteed here because significance filtering truncates effect size variability and inflates the average effect size.

Another issue with this filtering approach is that non-significant results are not only excluded, they are also incorrectly equated with no effect (Cohen, 1994; Nickerson, 2000; Wasserstein and Lazar, 2016). As Figure 3 illustrates, non-significance does not necessarily correspond to point estimated effect sizes of zero. Moreover, not shown in the graph due to clutter, there is high uncertainty in most effects due to small sample sizes: for example, *N* = 24 and *p* = 0.11 produces a point-estimated effect size in the medium range (*r* = 0.33) with a wide confidence interval (95% CI [−0.08, 0.65]).

### Comparison of Significance Filtered Means Versus Means of As-Reported Effects

**FIGURE 4:**
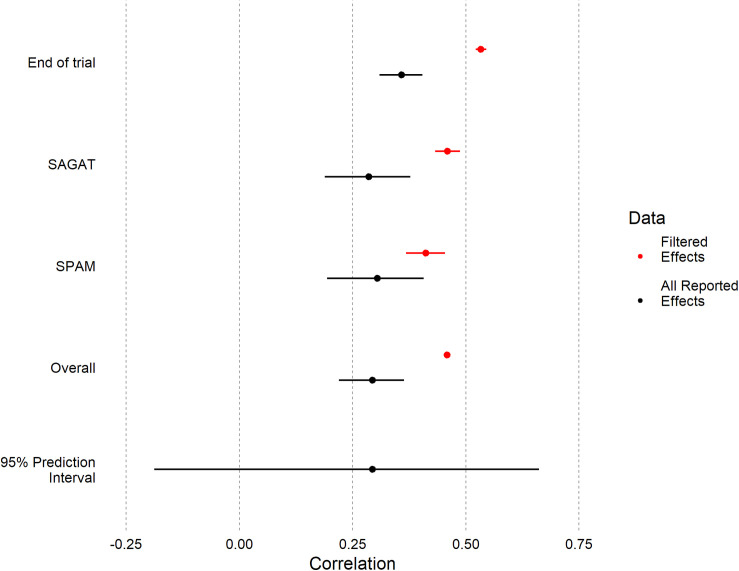
Forest plot depicting mean correlations between SA and performance, both overall and for individual SA measures. Meta-analytic means and prediction interval using all 241 reported effects are shown in black; means of filtered effects (Table 1) are shown in red. For the reason previously described in 2.1, no confidence interval could be calculated for the overall filtered mean.

In order to determine the impact of significance filtering on the means reported in Endsley (2019), we compared these filtered means (see section “Dataset” and Table 1) to means from a meta-analytic model using all effects (see Figure 4). To make the variance in effects more stable, we *z*-transformed correlations and calculated their variance using Fisher’s *Z* (Cooper et al., 2009; Hafdahl and Williams, 2009). Next, we used these values in a multivariate multilevel meta-analytic model (e.g., Assink and Wibbelink, 2016) to account for the multiple, repeated dependent measures nested in papers (two papers contained data from multiple experiments, these were treated as separate studies). In addition, meta-analytic models were fit using cluster robust variance estimation to adjust for small sample sizes and the unknown dependencies in sampling error among correlations from the same paper (Pustejovsky and Tipton, 2018). The *z*-values and confidence intervals were transformed back to correlation coefficients in the reported results. We used this more complex model because averaging effects requires making assumptions that are unlikely to hold and will often remove useful information (Borenstein et al., 2009; Scammacca et al., 2014). For full details about the analyses with meta-analytic models see Bakdash et al. (2020c).

The overall filtered mean (*r* = 0.46) was 56% higher (Type M error = 1.56) than the overall meta-analytic mean for all effects (*r* = 0.29). Likewise, we found similar patterns for the End of Trial and SAGAT measure. For SPAM, the magnitudes of the filtered and meta-analytic model means were closer with partially overlapping confidence intervals.

To evaluate uncertainty across all as-reported effects, we calculated the prediction interval^9^ : the plausible distribution for all individual effects (Borenstein et al., 2009). For all effects, the 95% prediction interval had an enormous range [−0.19, 0.66]. This interval includes non-random or systematic variation in the (estimated) distribution of true effects commonly referred to as meta-analytic heterogeneity, as opposed to only the random variation in effects due to sampling error (Borenstein et al., 2009). Here, variance among true effects approached a medium effect size ($\hat{\tau }$ = 0.24; see Table 2), nearing the magnitude of the mean overall effect from the correct meta-analytic model (*r* = 0.29). This heterogeneity was non-trivial both between and within papers, indicating the uncertainty in true effects was not solely due to differences among papers/datasets (e.g., experiment design, domain, or task). It was not possible to assess heterogeneity on significance filtered effects because they were reported as averages in Endsley (2019).

**TABLE 2:** All reported effects: Meta-analytic model parameters.

Parameter	Estimated value [95% CI]
*$\hat{\tau }$* = Standard deviation of true effects (due to total heterogeneity), interpret as *r* value	0.24 [0.19, 0.30]
*${\hat{\tau }}_{1}$* = Standard deviation of true effects (due to between-paper heterogeneity), interpret as *r* value	0.21 [0.16, 0.28]
*${\hat{\tau }}_{2}$* = Standard deviation of true effects (due to within-paper heterogeneity), interpret as *r* value	0.11 [0.08, 0.14]
*I^2^* = Index of dispersion: Variance due to heterogeneity relative to total variance	70.67% [56.83%, 81.26%]

#### Proportion of Effects Below/Above Their Filtered Means

To further evaluate the distribution of as-reported effects compared to filtered means, we also quantified the proportion of effects below meaningful thresholds using a recently developed method (Mathur and VanderWeele, 2019, 2020); see Supplementary Material 1.7 for details. This technique is not a filtering or vote-counting method, but rather, it provides quantitative insights into the distributions of effects by evaluating the proportions below/above specified thresholds.

The proportion analysis showed the overall filtered mean (*r* = 0.46) was a vast overestimate; 92% of effects were below it. Expressed as a natural frequency (Gigerenzer et al., 2007), about 222 out of the 241 individual effects were lower than the overall filtered threshold. Similar amounts of overestimation were found for each of the three SA measures (see Supplementary Material 1.7, Figures 4, 5).

## Discussion and Limitations

We have shown that selection using significance in a meta-analytic context produces a considerable distortion that is unrepresentative of all as-reported results, exaggerating the magnitude of meta-analytic mean effects. Using all effects as reported for SA-performance associations, we found meta-analytic effect sizes in approximately the medium range compared to the large range for significance filtering: This was a 1.56 times exaggeration or Type M error in the overall mean effect size. Moreover, 92% of the as-reported effects were below the overall significance filtered mean, indicating that it is highly biased upward. In general, selection using *p*-values impedes falsification (Kriegeskorte et al., 2009; Vul et al., 2009; Yarkoni, 2009; Ioannidis et al., 2014; Wasserstein and Lazar, 2016; Nelson et al., 2018). Popper (1962) expresses “It is easy to obtain confirmation, or verification, for nearly every theory—if we look for confirmations …. Every genuine test of a theory is an attempt to falsify it, or refute it.” (p. 36). Clever demonstrations of obviously specious results, boundary conditions where the null is actually true, supported using significance filtering, exemplify why it is problematic for falsification (Bennett et al., 2011; Simmons et al., 2011).

The fact that a high percentage of papers, most with multiple comparisons, can achieve *at-least-one* statistically significant result is insufficient evidence for drawing any broader meta-analytic conclusions. In small samples, significant effects appear especially impressive because they seem difficult to achieve due to noise and have striking magnitudes; this leads to a widespread but erroneous belief that such results are robust, common, and hence likely to replicate (Gelman and Carlin, 2014; Loken and Gelman, 2017). In general, significance filtered results are likely to be exaggerated (e.g., Vul et al., 2009; Yarkoni, 2009; Simmons et al., 2011; Button et al., 2013; Gelman and Carlin, 2014; Loken and Gelman, 2017; Vasishth et al., 2018; Bishop, 2020a, b) and hence are unlikely to replicate (Munafò et al., 2017).

In a meta-analytic context, the consequences of significance filtering are especially severe because this practice will distort the broader evidence provided with a quantitative synthesis (Vosgerau et al., 2019). In fact, Glass (2015) noted that he invented meta-analysis to counter this very issue and related problems. Given that significance filtering contradicts the very purpose of meta-analysis (Button et al., 2013; Glass, 2015; Vosgerau et al., 2019), we assert that Endsley (2019) is not a meta-analysis. Critically, even traditional vote-counting is no longer considered an appropriate meta-analytic technique for synthesizing average effect sizes (Borenstein et al., 2009; Gurevitch et al., 2018; Higgins et al., 2019; Mathur and VanderWeele, 2019). Rather than using *p*-values or other outcome-dependent criteria to select results, the point of meta-analysis is to use all available pertinent information from papers and results based on a systematic review with specified *a priori* inclusion/exclusion criteria.

Issues with significance filtering and other selective inclusion/reporting of results have been attributed in part to cognitive biases (Bishop, 2020b) and are widely recognized as problematic in multiple fields (Bishop, 2019), including psychology (Simmons et al., 2011), neuroscience (Kriegeskorte et al., 2009; Vul et al., 2009), and health science research (Goldacre, 2010, 2014). The American Statistical Association’s statement on *p*-values unequivocally stipulates scientific conclusions should not rely on bright-line thresholds for *p*-values, nor should selective analyses be performed based on *p*-values:

“Practices that reduce data analysis or scientific inference to mechanical ‘bright-line’ rules (such as ‘*p* < 0.05’) for justifying scientific claims or conclusions can lead to erroneous beliefs and poor decision making. A conclusion does not immediately become ‘true’ on one side of the divide and ‘false’ on the other…

A *p*-value, or *statistical significance, does not measure the size of an effect or the importance of a result* [emphasis added].”(Wasserstein and Lazar, 2016, pp. 131–132).

### Limitations

Although the analyses conducted and results reported here provide a compelling case about the negative consequences of significance filtering in a meta-analytic context, there are a number of limitations in our critique that should be noted. This work does not comprehensively evaluate Endsley (2019); instead we focus on SA-performance associations, which Endsley refers to as SA predictiveness. Endsley (2019) also evaluated SA sensitivity (differences in SA attributed to training, participant expertise) and SA intrusiveness (whether the method for assessing SA impacted performance or workload). While we did not examine sensitivity and intrusiveness in detail, we posit there are issues that are similar to our detailed evaluation of predictiveness. SA sensitivity also relied on the atypical *at-least-one* methodology that produced the predictiveness score. SA intrusiveness consisted of a narrative form of vote-counting using *p*-values, with non-significance also incorrectly equated with no effect. For both sensitivity and intrusiveness, it is possible that effect sizes were unavailable and could not be calculated from the information provided in papers. If exact *p*-values are available there are many techniques for synthesizing *p*-values that are superior to vote-counting (see Becker, 1994; Cinar and Viechtbauer, 2020). Nevertheless, if only significance or non-significance is reported in papers (rather than exact *p*-values) vote-counting will likely be the only option. Another limitation was that the papers and results we included here may have differed from Endsley (2019) for reasons other than significance filtering. In Endsley’s work, we identified issues with internal reproducibility of results using data directly from Endsley (Supplementary Material 1.8) as well as other inconsistencies that may be due to lack of specified inclusion/exclusion criteria (Supplementary Material 1.9). The main purpose of this work was a direct comparison of all effects as-reported to results in Endsley, not to reproduce significance filtering. Therefore, we used the filtered mean values as-reported or calculated from Endsley (see section “Dataset” and Table 1).

A clear limitation of our work is that it is not a meta-analysis; literature inclusion was based on papers in Appendix C, Endsley (2019) using our previously described minimal criteria (see section “Dataset”), rather than a systematic review. This work is a direct comparison between all as-reported effects versus significance filtered effects. Consequently, we do not address issues such as the file drawer problem (also known as publication bias; Rosenthal, 1979).

Similarly, some papers, included both here and by Endsley (2019), selectively reported only significant results; this is not a new issue (Hedges, 1984). Bishop and Thompson (2016) called omission of undesirable results ghost *p*-hacking; borrowing from their terminology, we use the term “ghost results” to describe SA-performance associations that ware clearly assessed but either not reported due to not meeting significance, or only reported as not meeting significance without details (*p*-value or effect size). By definition and for direct comparison, we only used (detailed) effects as-reported in the analyses here. Nevertheless, we coded ghost results (see the data dictionary in Bakdash et al., 2020d for details) and found they were pervasive: 139 ghost results in 14 papers. For an actual meta-analysis, we caution that not including ghost results (and publication bias) may lead to overestimates of effect sizes and underestimates of variance.

## Conclusion

We have shown there is a substantial difference between analyses with as-reported effects compared to analyses using significance filtered effects. With the considerable caveat our meta-analytic means were not based on a systematic review and did not take into account any ghost results, our results indicate a limited validity for SA and performance (medium mean effects, high systematic variance in true effects, and the majority of effects below their filtered means). This is in contrast to results using significance filtering, which indicate strong validity (approximately large [filtered] mean effects with minimal variance in confidence intervals). Our interpretation of limited validity is not consistent with most current SA theories. In addition, prior work has raised concerns about the potential unfalsifiability for testing SA theories (Dekker and Hollnagel, 2004); significance filtering amplifies concerns about falsifiability. If a particular theory can only be quantitatively tested by selecting supporting results while excluding less desirable or undesirable results, then the theory itself is unfalsifiable (Ferguson and Heene, 2012).

Evidence of limited validity has practical implications for associations among SA-performance effects. For example, using training and system design to increase SA is posited to also improve performance (Endsley and Jones, 2011). While the theorized causal relationship between SA and performance is debatable (Flach, 1995; Dekker and Hollnagel, 2004), improving SA is often a goal, SA is sometimes used as a proxy for performance, and SA is even occasionally operationalized as task performance. SA and performance are often assessed in real-world, safety-critical work environments such as aviation, driving, health care, and the military. Thus, using established meta-analytic methods to accurately quantify the magnitude, uncertainty, and distribution of SA-performance effects is essential.

The scientific methods for meta-analysis are well-established (Borenstein et al., 2009; Koricheva et al., 2013; Glass, 2015; Cooper et al., 2019). Other recommended practices go further, also recommending sharing data and code for quality control, reproducibility of results, and updating earlier meta-analyses (Button et al., 2013; Lakens et al., 2016; Gurevitch et al., 2018; Maassen et al., 2020; Polanin et al., 2020). However, even the well-established methods for research synthesis are not universally followed. Outdated methods such as traditional vote-counting and unweighted models are still commonly used in some fields; this has been attributed to a lack of training and knowledge (Koricheva et al., 2013). We have shown here that the unusual *at-least one* significance filtering method is even more problematic than outdated methods, because results are exaggerated by predetermined minimum effect sizes. In contrast, when research synthesis is conducted using modern, established methods, it provides: “a more objective, informative and powerful means of summarizing the results … compared to narrative/qualitative reviews and vote counting” (Koricheva et al., 2013, p. 13).

## Data Availability Statement

The datasets presented in this study can be found in online repositories. The names of the repository/repositories and accession number(s) can be found below: The data and code to reproduce the results can be found on the Open Science Framework: https://doi.org/10.17605/OSF.IO/BXPJC or our Code Ocean capsule: https://doi.org/10.24433/CO.1447674.v2 (pending updated version: https://doi.org/10.24433/CO.1447674.v2).

## Ethics Statement

The United States Army Research Laboratory’s Institutional Review Board approved this project as not human subjects research (ARL 19–219).

## Author Contributions

All authors contributed to writing and revising the manuscript. JB, LM, and EZ extracted and coded data from manuscript. JB and LM wrote the code for data cleaning and analyses.

## Conflict of Interest

The authors declare that the research was conducted in the absence of any commercial or financial relationships that could be construed as a potential conflict of interest.

## Footnotes

1 Selection using statistical significance, which we also refer to as significance filtering and filtering, has many other terms. It is also called the significance filter, data dredging, fishing, double dipping, cherry-picking, and *p*-hacking (Nuzzo, 2014; Aschwanden, 2019). Use of the term *p*-hacking has been criticized and is controversial, but the term is now widely used inside and even outside of science (Aschwanden, 2019).

2 Endsley (2019) states that this approach was used by Vidulich (2000), but this does not seem to be the case. While Vidulich performed vote-counting on papers with multiple effects, in each paper *all* effects were counted and then a single score was assigned using a mini vote-count method (i.e., if the majority of effects in a paper were significant, the entire paper [and/or specific measure in the paper] was scored as +1, etc.). That is, Vidulich did *not* use an at-least-one, directional vote-count. At the time Vidulich was published, the year 2000, vote-counting was still widely used. In support of the predictiveness score, Endsley (2019) also cites Wickens (1998). However, we contend that Wickens makes a compelling case for interpreting *p*-values along a continuum rather than using statistical significance as an (arbitrary) cut-off.

3 We focus here on filtering using statistical significance, but circular logic for selection can be easily applied to other statistical methods and estimators (see Gelman, 2016): such as filtering using effect size magnitude, cross-validation, Bayes factors, etc.

4 Note that the means taken from Endsley’s Table 5 presumably use data from Endsley, Appendix C. However, we were unable to use Endsley, Appendix C data to reproduce most of the mean values in Endsley, Table 5 (see Supplementary Material 1.8). Also, for 11 out of 46 papers, Endsley included predictiveness scores but not the mean correlations; the reasons for this were generally unknown (see Supplementary Material 1.9). Nevertheless, using only the subset of 38 papers we included to select data from Endsley, Appendix C yields similar values to our Table 1 (and Endsley, Table 5): End of Trial: *r* = 0.53, SAGAT: *r* = 0.46, SPAM: *r* = 0.43, and Overall: *r* = 0.45.

5 Using the averaging method described in Endsley (2019), significance filtered effects in our dataset had a slightly lower (*r* = 0.43) filtered overall mean. We found evidence that results were not just significance filtered (see section “Limitations”), but we were unable to address this beyond identifying reproducibility issues (Supplementary Material 1.8) and a lack of clear inclusion/exclusion criteria (Supplementary Material 1.9).

6 We caution that a point estimate of post-hoc statistical power generally has enormous uncertainty, see Yuan and Maxwell (2005). The calculation of statistical power is only used as an example for a simulated predictiveness score, it should not be interpreted as veridical estimate of power. Furthermore, this simplified power calculation does not address effect size or meta-analytic heterogeneity (McShane and Böckenholt, 2014), which was substantial here (see section “Comparison of Significance Filtered Means versus Means of As-Reported Effects”).

7 We use the median *N* instead of the mean *N* because it is a more conservative estimate for power and because it is more representative of sample size for the included papers given the high skew (see Supplementary Material 1.4, Figure 1).

8 Typically, instead of power, α = 0.05 is used for the Type I error rate.

9 “The confidence interval quantifies the accuracy of the mean, while the prediction interval addresses the actual dispersion of [individual] effect sizes, and the two measures are not interchangeable.” (Borenstein et al., 2009, p. 131).
