# Supplementary material for: Statistical Significance Filtering Overestimates Effects and Impedes Falsification: A Critique of Endsley (2019)
Source: Front Psychol. 2020 Dec 22;11:609647. doi: 10.3389/fpsyg.2020.609647 (PMC7783317; doi:10.3389/fpsyg.2020.609647)
Supplement: Supplementary file 1 [file Data_Sheet_1.docx]

# Supplementary Material

## Excluded Papers

A total of eight papers were completely excluded with one paper partially excluded, see Table 1.

***Table 1.*** Exclusion reasons with explanations with the corresponding number of papers and their references. For citations to papers in the references see Endsley (2019).

| *Exclusion Reason* | *Number of Papers* | *Explanation* | *References* |
| --- | --- | --- | --- |
| No SA-performance association | 7 | In Chauvin et al. (2008), decisions are subjective, thus it is unclear what constitutes good or bad performance. | Chauvin et al. (2008) |
|  |  | In Li et al. (2007), no association for SA and performance was reported. SA was assessed for two different displays, along with trust and confidence (this could also be categorized as no measure of performance). | Li et al. (2007) |
|  |  | In McGowan and Banbury (2004), hazard perception using anticipation is treated as performance in Endsley. Anticipating hazards is primarily a measure of attention and perception, essentially SA, rather than a measure of driving performance (e.g., speed, lane deviations, following distance, collisions). In fact the original authors describe the hazard perception test as “a dependent measure of SA” (McGowan and Banbury, p. 292). Thus, the correlation reported here appears to be between two measures of SA. | McGowan and Banbury (2004) |
|  |  | In McKenna et al. (2014), an SA-performance association is reported as *p* ≤ 0.01 with no effect size. | McKenna et al. (2014) |
|  |  | Endsley (2019) codes the mean effect in Sethumadhavan (2011) as *0.488*. However, this is a mean value: "Tukey’s HSD analysis on SA showed that participants in the information acquisition condition (*M* = 0.488, *SD* = 0.119)" (p. 4). Sethumadhavan does not report any SA-performance associations (this could also be categorized as no measure of performance). | Sethumadhavan (2011) |
|  |  | In Soliman (2010), no association for SA and performance was reported. | Soliman (2010) |
|  |  | In Strybel et al. (2009), to evaluate SA and performance relationships, pseudo-R2 effect sizes are reported from a hierarchical linear model (HLM) or multilevel model (MLM). There are numerous ways to calculate HLM/MLM pseudo effect sizes, but there is no clear conversion to a correlation/simple regression effect size. | Strybel et al. (2009) |
| Insufficient data | 2 | The real-time probe and SALSA were only assessed in a single paper each. This was insufficient data to fit a multilevel meta-analytic model. For Jones and Endsley (2004), only the real-time probe was excluded; the SAGAT was still included here. | Jones and Endsley (2004): Partial exclusion, real-time probe  Kaber et al. (2006): SALSA measure of SA |

## Additional Information about Included Papers

Twelve of the papers (two partially) we included here would not have met our prior systematic review criteria (Bakdash et al. 2020c) due to model overfitting or SA and performance in teams instead of individuals (see Table 2). The numbers in Table 2 add to 12, instead of 13, because one paper had both model overfitting and teams.

***Table 2.*** Caveats about included papers with overfitting and teams. For citations to references see Endsley (2019)

| *Caveat* | *Number of Papers* | *Explanation* | *References* | |  |
| --- | --- | --- | --- | --- | --- |
| Model overfitting | 10 | In these papers, SA and performance were assessed multiple times for each participant with the data incorrectly analyzed as independent. For example, Strybel et al. (2008) describes an experiment with a sample size of *N* = 13 but reports *r*(52) = 0.32, *p* = 0.02 (p. 4). That is, 52 degrees of freedom. However, the degrees of freedom for a Pearson correlation coefficient is (*N* – 2) so there should be 11 degrees of freedom (13 – 2). Moreover, with *N* = 13 the effect size of *r* = 0.32 would be non-significant (even if one-tailed).  Both Puushka et al. (2018) and Strybel et al. (2008) have some overfit results, but not all results.  Note there was also model overfitting in Kaber et al. (2006), but it was previously excluded because it used the SALSA which was unique to that work. | | Bacon and Strybel (2013)  Endsley et al. (2000): *Cited here as Jones and Endsley (2004)*  Endsley (1990)  Ikuma et al. (2014)  Kaber et al. (2016)  Ma and Kaber (2005)  Puushka et al. (2018)  Strybel et al. (2008)  Strybel et al. (2013)  Zhang et al. (2010) | |
| Teams | 3 | It can be highly informative to look at both the individual and team-levels (Hackman, 2003), but we contend that for an actual meta-analysis it is problematic to mix the two levels of analysis together.  McKenna et al. (2014) also assessed SA and performance in teams, but was previously excluded (see Supplemental Material 1.1). | | Endsley (1990): *Also included above*  Hamilton et al. (2017)  Prince et al. (2017) | |

Note six papers with overfit results (Endsley et al. 2000; Kaber et al. 2006; Kaber et al. 2016; Ma and Kaber, 2005; Strybel 2008; Zhang et al. 2010) were erroneously included in Bakdash et al. (2020a; 2020b; 2020c). The mistakes for including papers with overfit results demonstrate that the sample size and degrees of freedom should be carefully checked as part of formal inclusion criteria for a systematic review; and even more importantly, prior to publication, by the authors, reviewers, and editor. The incorrectly included overfit papers will be removed from the next version of our unpublished meta-analysis.

## References for the 38 Included Papers

Bacon, L. P., & Strybel, T. Z. (2013). Assessment of the validity and intrusiveness of online-probe questions for situation awareness in a simulated air-traffic-management task with student air-traffic controllers. *Safety Science*, *56*, 89–95. <https://doi.org/10.1016/j.ssci.2012.06.019>

Cooper, S., Kinsman, L., Buykx, P., McConnell-Henry, T., Endacott, R., & Scholes, J. (2010). Managing the deteriorating patient in a simulated environment: Nursing students knowledge, skill and situation awareness. *Journal of Clinical Nursing*, *19*(15–16), 2309–2318. <https://doi.org/10.1111/j.1365-2702.2009.03164.x>

Cummings, M. L., & Guerlain, S. (2007). Developing Operator Capacity Estimates for Supervisory Control of Autonomous Vehicles. *Human Factors*, *49*(1), 1–15. <https://doi.org/10.1518/001872007779598109>

Durso, F. T., Bleckley, M. K., & Dattel, A. R. (2006). Does Situation Awareness Add to the Validity of Cognitive Tests? *Human Factors: The Journal of the Human Factors and Ergonomics Society*, *48*(4), 721–733. <https://doi.org/10.1518/001872006779166316>

Durso, Francis T., Hackworth, C. A., Truitt, T. R., Crutchfield, J., Nikolic, D., & Manning, C. A. (1998). Situation Awareness as a Predictor of Performance for En Route Air Traffic Controllers. *Air Traffic Control Quarterly*, *6*(1), 1–20. <https://doi.org/10/gf36hf>

Endsley, M. R. (1990). Predictive utility of an objective measure of situation awareness. *Proceedings of the Human Factors and Ergonomics Society Annual Meeting*, *34*, 41–45. <http://pro.sagepub.com/content/34/1/41.short>

*Endsley, M. R., Sollenberger, R. L., Nakata, A., & Stein, E. S. (2000). *Situation Awareness in Air Traffic Control: Enhanced Displays for Advanced Operations* (Technical Note DOT/FAA/CT-TN00/01). <http://hf.tc.faa.gov/publications/2000-situation-awareness-in-air-traffic-control/full_text.pdf>

Entin, E. B. (2000). An Exploratory Investigation of Relationships between Situation Awareness and Performance in an Attack Helicopter Domain. *Proceedings of the Human Factors and Ergonomics Society Annual Meeting*, *44*, 113–115. <https://doi.org/10.1177/154193120004400130>

Gatsoulis, Y., Virk, G. S., & Dehghani-Sanij, A. A. (2010). On the measurement of situation awareness for effective human-robot interaction in teleoperated systems. *Journal of Cognitive Engineering and Decision Making*, *4*(1), 69–98. <https://doi.org/10.1518/155534310X495591>

Gugerty, L. J. (1997). Situation awareness during driving: Explicit and implicit knowledge in dynamic spatial memory. *Journal of Experimental Psychology: Applied*, *3*(1), 42–66. <https://doi.org/10.1037/1076-898X.3.1.42>

Gutzwiller, R. S., & Clegg, B. A. (2013). The role of working memory in levels of situation awareness. *Journal of Cognitive Engineering and Decision Making*, *7*(2), 141–154. <https://doi.org/10.1177/1555343412451749>

Hamilton, K., Mancuso, V., Mohammed, S., Tesler, R., & McNeese, M. (2017). Skilled and Unaware: The Interactive Effects of Team Cognition, Team Metacognition, and Task Confidence on Team Performance. *Journal of Cognitive Engineering and Decision Making*, *11*(4), 382–395. <https://doi.org/10.1177/1555343417731429>

Hogan, M. P., Pace, D. E., Hapgood, J., & Boone, D. C. (2006). Use of human patient simulation and the situation awareness global assessment technique in practical trauma skills assessment. *The Journal of Trauma*, *61*(5), 1047–1052. <https://doi.org/10.1097/01.ta.0000238687.23622.89>

Ikuma, L. H., Harvey, C., Taylor, C. F., & Handal, C. (2014). A guide for assessing control room operator performance using speed and accuracy, perceived workload, situation awareness, and eye tracking. *Journal of Loss Prevention in the Process Industries*, *32*, 454–465. <https://doi.org/10.1016/j.jlp.2014.11.001>

Jannat, M., Hurwitz, D. S., Monsere, C., & Funk, K. H. (2018). The role of driver’s situational awareness on right-hook bicycle-motor vehicle crashes. *Safety Science*, *110*, 92–101. <https://doi.org/10.1016/j.ssci.2018.07.025>

Jipp, M., & Ackerman, P. L. (2016). The Impact of Higher Levels of Automation on Performance and Situation Awareness: A Function of Information-Processing Ability and Working-Memory Capacity. *Journal of Cognitive Engineering and Decision Making*, *10*(2), 138–166. <https://doi.org/10.1177/1555343416637517>

Kaber, D. B., & Endsley, M. R. (2004). The effects of level of automation and adaptive automation on human performance, situation awareness and workload in a dynamic control task. *Theoretical Issues in Ergonomics Science*, *5*(2), 113–153. <https://doi.org/10.1080/1463922021000054335>

Kaber, D., Jin, S., Zahabi, M., & Pankok, C. (2016). The effect of driver cognitive abilities and distractions on situation awareness and performance under hazard conditions. *Transportation Research Part F: Traffic Psychology and Behaviour*, *42*, 177. <https://doi.org/10.1016/j.trf.2016.07.014>

Kass, S. J., VanWormer, L. A., Mikulas, W. L., Legan, S., & Bumgarner, D. (2011). Effects of mindfulness training on simulated driving: Preliminary results. *Mindfulness*, *2*(4), 236–241. <https://doi.org/10.1007/s12671-011-0066-1>

Kraemer, J., & Süß, H.-M. (2015). Real Time Validation of Online Situation Awareness Questionnaires in Simulated Approach Air Traffic Control. *Procedia Manufacturing*, *3*, 3152–3159. <https://doi.org/10.1016/j.promfg.2015.07.864>

Lo, J. C., Sehic, E., Brookhuis, K. A., & Meijer, S. A. (2016). Explicit or implicit situation awareness? Measuring the situation awareness of train traffic controllers. *Transportation Research Part F: Traffic Psychology and Behaviour*, *43*, 325–338. <https://doi.org/10.1016/j.trf.2016.09.006>

Loft, S., Bowden, V., Braithwaite, J., Morrell, D. B., Huf, S., & Durso, F. T. (2015). Situation Awareness Measures for Simulated Submarine Track Management. *Human Factors: The Journal of the Human Factors and Ergonomics Society*, *57*(2), 298–310. <https://doi.org/10.1177/0018720814545515>

Loft, S., Jooste, L., Li, Y. R., Ballard, T., Huf, S., Lipp, O. V., & Visser, T. A. W. (2018). Using Situation Awareness and Workload to Predict Performance in Submarine Track Management: A Multilevel Approach. *Human Factors: The Journal of the Human Factors and Ergonomics Society*, *60*(7), 978–991. <https://doi.org/10.1177/0018720818784803>

Loft, S., Morrell, D. B., & Huf, S. (2013). Using the situation present assessment method to measure situation awareness in simulated submarine track management. *International Journal of Human Factors and Ergonomics*, *2*(1), 33. <https://doi.org/10.1504/IJHFE.2013.055975>

Loft, S., Morrell, D. B., Ponton, K., Braithwaite, J., Bowden, V., & Huf, S. (2016). The Impact of Uncertain Contact Location on Situation Awareness and Performance in Simulated Submarine Track Management. *Human Factors*, *58*(7), 1052–1068. <https://doi.org/10.1177/0018720816652754>

Ma, R., & Kaber, D. B. (2005). Situation awareness and workload in driving while using adaptive cruise control and a cell phone. *International Journal of Industrial Ergonomics*, *35*(10), 939–953. <https://doi.org/10.1016/j.ergon.2005.04.002>

McDermott, P. L., & Fisher, A. (2013). Methodologies for Assessing Situation Awareness of Unmanned System Operators. *Proceedings of the Human Factors and Ergonomics Society Annual Meeting*, *57*, 167–171. <https://doi.org/10.1177/1541931213571038>

O’Brien, K. S., & O’Hare, D. (2007). Situational awareness ability and cognitive skills training in a complex real-world task. *Ergonomics*, *50*(7), 1064–1091. <https://doi.org/10.1080/00140130701276640>

Paletta, L., Dini, A., Murko, C., Yahyanejad, S., Schwarz, M., Lodron, G., Ladstätter, S., Paar, G., & Velik, R. (2017). Towards Real-time Probabilistic Evaluation of Situation Awareness from Human Gaze in Human-Robot Interaction. *Proceedings of the Companion of the 2017 ACM/IEEE International Conference on Human-Robot Interaction - HRI ’17*, 247–248. <https://doi.org/10.1145/3029798.3038322>

Pierce, R. S., Strybel, T. Z., & Vu, K.-P. L. (2008). Comparing situation awareness measurement techniques in a low fidelity air traffic control simulation. *Proceedings of the 26th International Congress of the Aeronautical Sciences (ICAS), Anchorage, AS*.

Prince, C., Ellis, E., Brannick, M. T., & Salas, E. (2007). Measurement of Team Situation Awareness in Low Experience Level Aviators. *The International Journal of Aviation Psychology*, *17*(1), 41–57. <https://doi.org/10.1080/10508410709336936>

Puuska, S., Rummukainen, L., Timonen, J., Lääperi, L., Klemetti, M., Oksama, L., & Vankka, J. (2018). Nationwide critical infrastructure monitoring using a common operating picture framework. *International Journal of Critical Infrastructure Protection*, *20*, 28–47. <https://doi.org/10.1016/j.ijcip.2017.11.005>

Salmon, P. M., Stanton, N. A., Walker, G. H., Jenkins, D., Ladva, D., Rafferty, L., & Young, M. (2009). Measuring Situation Awareness in complex systems: Comparison of measures study. *International Journal of Industrial Ergonomics*, *39*(3), 490–500. <https://doi.org/10.1016/j.ergon.2008.10.010>

Stanners, M., & French, H. T. (2005). *An Empirical Study of the Relationship between Situation Awareness and Decision Making* (DSTO-TR-1687). DEFENCE SCIENCE AND TECHNOLOGY ORGANIZATION EDINBURGH (AUSTRALIA) LAND OPERATIONS DIV. <https://apps.dtic.mil/docs/citations/ADA434593>

Strybel, Thomas Z., Vu, K.-P. L., Kraft, J., & Minakata, K. (2008). *Assessing the Situation Awareness of Pilots Engaged in Self Spacing*. Proceedings of the Human Factors and Ergonomics Society Annual Meeting, *52*(1), 11–15. <https://doi.org/10/fzs5gq>

Strybel, T.Z., Vu, K.-P. L., Battiste, V., & Johnson, W. (2013). Measuring the Impact of NextGen Operating Concepts for Separation Assurance on Pilot Situation Awareness and Workload. *International Journal of Aviation Psychology*, *23*, 1–26. <https://doi.org/10/ggqf7q>

Sulistyawati, K., Wickens, C. D., & Chui, Y. P. (2011). Prediction in Situation Awareness: Confidence Bias and Underlying Cognitive Abilities. *International Journal of Aviation Psychology*, *21*(2), 153–174. <https://doi.org/10.1080/10508414.2011.556492>

Zhang, T., Kaber, D., & Hsiang, S. (2010). Characterisation of mental models in a virtual reality-based multitasking scenario using measures of situation awareness. *Theoretical Issues in Ergonomics Science*, *11*(1–2), 99–118. <https://doi.org/10.1080/14639220903010027>

*Note Endsley (2019) cites and uses data from Jones and Endsley (2004), which contains the same experiment and data as Endsley (2000) cited above. In Endsley (2000) the term on-line probe is used. Whereas, in Jones and Endsley (2004) this is called a real-time probe (both appear to measure response time to SAGAT questions). We were unable to include the on-line/real-time probe measure because it was only used in one paper. Also, Jones and Endsley (2004) reports a second experiment with 5 teams. We did not include the second experiment because it was not listed in Endsley (2019), Appendix C.

## Histograms of Included Papers: Sample Sizes and Number of Effects

For papers we include, the two figures below, respectively, show the distributions of sample sizes and the number of effects. Note data in both figures is positively skewed; this is also shown with the median values being lower than the mean values.

**Figure 1.**
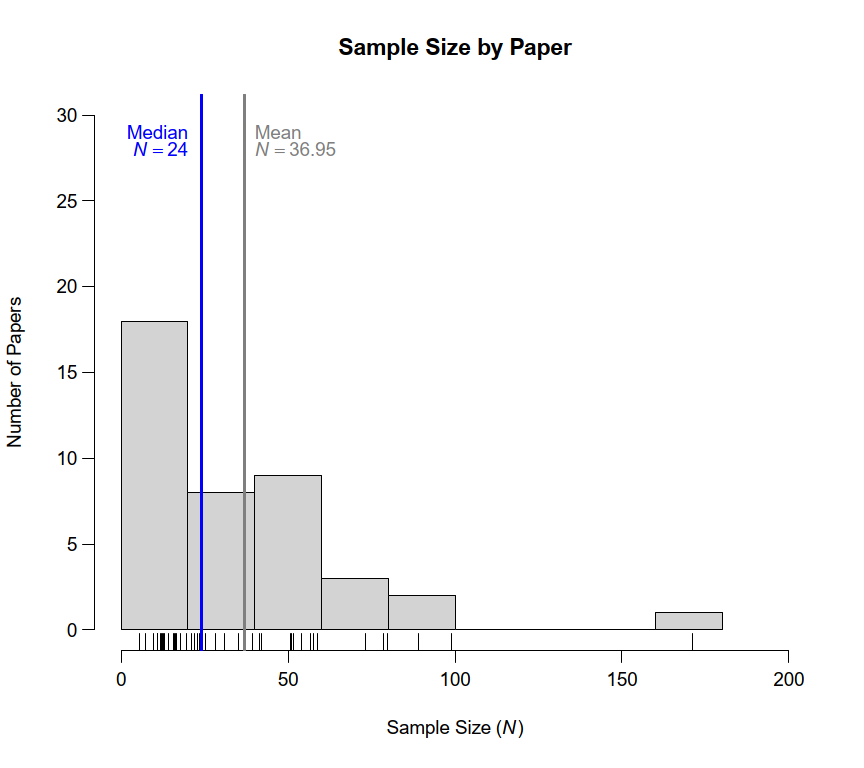


Histogram of sample size by paper (two papers contributed multiple studies). The x-axis is sample size, with tick marks representing individual papers (randomly jittered by 1.25 to improve visibility). The y-axis is the number of papers. The vertical lines depict the median and mean sample sizes.

**Figure 2**
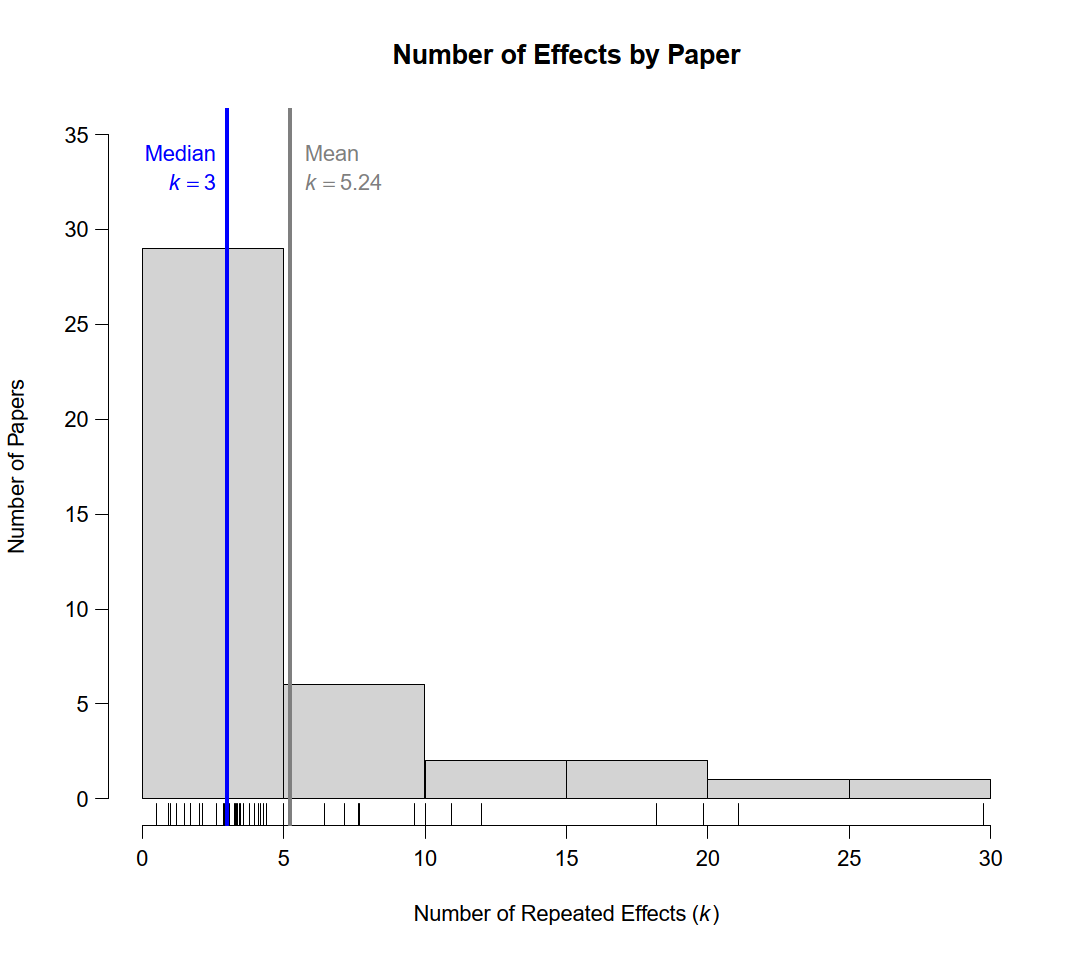
 Histogram of number of effects by paper. The x-axis is the number of effects in each paper, with tick marks representing individual papers (randomly jittered by 0.5 to improve visibility). The y-axis is the number of papers. The vertical lines depict the median and mean number of repeated effects per paper.

## R Package References

Aust, F., & Barth, M. (2020). *papaja: Create APA manuscripts with R Markdown* [Manual]. <https://github.com/crsh/papaja>

Bates, D., & Maechler, M. (2019). *Matrix: Sparse and dense matrix classes and methods* [Manual]. <https://CRAN.R-project.org/package=Matrix>

Boessenkool, B. (2020). *berryFunctions: Function collection related to plotting and hydrology* [Manual]. <https://CRAN.R-project.org/package=berryFunctions>

Champely, S. (2020). *pwr: Basic functions for power analysis* [Manual]. <https://CRAN.R-project.org/package=pwr>

Csárdi, G. (2019). *pkgconfig: Private configuration for “r” packages* [Manual]. <https://CRAN.R-project.org/package=pkgconfig>

Davison, A. C., & Hinkley, D. V. (1997). *Bootstrap methods and their applications*. Cambridge University Press. <http://statwww.epfl.ch/davison/BMA/>

Fisher, Z., Tipton, E., & Zhipeng, H. (2017). *robumeta: Robust variance meta-regression* [Manual]. <https://CRAN.R-project.org/package=robumeta>

Fox, J., & Weisberg, S. (2019). *An R companion to applied regression* (3rd ed.). Sage. <https://socialsciences.mcmaster.ca/jfox/Books/Companion/>

Fox, J., Weisberg, S., & Price, B. (2020). *carData: Companion to applied regression data sets* [Manual]. <https://CRAN.R-project.org/package=carData>

Gaslam, B. (2020). *fansi: ANSI control sequence aware string functions* [Manual]. <https://CRAN.R-project.org/package=fansi>

Genz, A., & Bretz, F. (2009). *Computation of multivariate normal and t probabilities*. Springer-Verlag.

Grosser, M. (2019). *snakecase: Convert strings into any case* [Manual]. <https://CRAN.R-project.org/package=snakecase>

Hallman, J. (2020). *tis: Time indexes and time indexed series* [Manual]. <https://CRAN.R-project.org/package=tis>

Hankin, R. K. S. (2006). Special functions in R: introducing the gsl package. *R News*, *6*(4).

Harrell Jr, F. E., Dupont, with contributions from C., & others., many. (2020). *Hmisc: Harrell miscellaneous* [Manual]. <https://CRAN.R-project.org/package=Hmisc>

Henry, L., & Wickham, H. (2020a). *purrr: Functional programming tools* [Manual]. <https://CRAN.R-project.org/package=purrr>

Henry, L., & Wickham, H. (2020b). *rlang: Functions for base types and core r and “tidyverse” features* [Manual]. <https://CRAN.R-project.org/package=rlang>

Henry, L., Wickham, H., & Chang, W. (2020). *ggstance: Horizontal “ggplot2” components* [Manual]. <https://CRAN.R-project.org/package=ggstance>

Hothorn, T. (2019). *TH.data: TH’s data archive* [Manual]. <https://CRAN.R-project.org/package=TH.data>

Hothorn, T., Bretz, F., & Westfall, P. (2008). Simultaneous inference in general parametric models. *Biometrical Journal*, *50*(3), 346–363. <https://doi.org/10/b5f2gc>

Hoyt, A. C. D. R. & W. T. (2014). *MAd: Meta-analysis with mean differences* [Manual]. <https://cran.r-project.org/package=MAd>

J, L. (2006). Plotrix: A package in the red light district of R. *R-News*, *6*(4), 8–12.

Lüdecke, D. (2019). *esc: Effect size computation for meta analysis (version 0.5.1)* [Manual]. <https://doi.org/10.5281/zenodo.1249218>

Mahmoudian, M. (2020). *varhandle: Functions for robust variable handling* [Manual]. <https://CRAN.R-project.org/package=varhandle>

Mathur, M. B., Wang, R., & VanderWeele, T. J. (2019). *MetaUtility: Utility functions for conducting and interpreting meta-analyses* [Manual]. <https://CRAN.R-project.org/package=MetaUtility>

Müller, K. (2018). *bindrcpp: An “rcpp” interface to active bindings* [Manual]. <https://CRAN.R-project.org/package=bindrcpp>

Müller, K., & Wickham, H. (2020). *tibble: Simple data frames* [Manual]. <https://CRAN.R-project.org/package=tibble>

Neuwirth, E. (2014). *RColorBrewer: ColorBrewer palettes* [Manual]. <https://CRAN.R-project.org/package=RColorBrewer>

Novomestky, F. (2012). *matrixcalc: Collection of functions for matrix calculations* [Manual]. <https://CRAN.R-project.org/package=matrixcalc>

Pustejovsky, J. (2020). *clubSandwich: Cluster-robust (sandwich) variance estimators with small-sample corrections* [Manual]. <https://CRAN.R-project.org/package=clubSandwich>

R Core Team. (2020). *R: A language and environment for statistical computing* [Manual]. <https://www.R-project.org/>

Revelle, W. (2020). *psych: Procedures for psychological, psychometric, and personality research* [Manual]. <https://CRAN.R-project.org/package=psych>

Rinker, T. W., & Kurkiewicz, D. (2018). *pacman: Package management for R* [Manual]. <http://github.com/trinker/pacman>

Sarkar, D. (2008). *Lattice: Multivariate data visualization with r*. Springer. <http://lmdvr.r-forge.r-project.org>

Schafer, J., Opgen-Rhein, R., Zuber, V., Ahdesmaki, M., Silva, A. P. D., & Strimmer., K. (2017). *corpcor: Efficient estimation of covariance and (partial) correlation* [Manual]. <https://CRAN.R-project.org/package=corpcor>

Soetaert, K. (2018). *shape: Functions for plotting graphical shapes, colors* [Manual]. <https://CRAN.R-project.org/package=shape>

Stephens, J., Simonov, K., Xie, Y., Dong, Z., Wickham, H., Horner, J., reikoch, Beasley, W., O’Connor, B., & Warnes, G. R. (2020). *yaml: Methods to convert r data to YAML and back* [Manual]. <https://CRAN.R-project.org/package=yaml>

Terry M. Therneau, & Patricia M. Grambsch. (2000). *Modeling survival data: Extending the Cox model*. Springer.

Tierney, L., Rossini, A. J., Li, N., & Sevcikova, H. (2018). *snow: Simple network of workstations* [Manual]. <https://CRAN.R-project.org/package=snow>

Venables, W. N., & Ripley, B. D. (2002). *Modern applied statistics with s* (4th ed.). Springer. <http://www.stats.ox.ac.uk/pub/MASS4>

Viechtbauer, W. (2010). Conducting meta-analyses in R with the metafor package. *Journal of Statistical Software*, *36*(3), 1–48. <https://doi.org/10/gckfpj>

Wei, T., & Simko, V. (2017). *R package “corrplot”: Visualization of a correlation matrix* [Manual]. <https://github.com/taiyun/corrplot>

Wickham, H. (2016). *ggplot2: Elegant graphics for data analysis*. Springer-Verlag New York. <https://ggplot2.tidyverse.org>

Wickham, H. (2019). *stringr: Simple, consistent wrappers for common string operations* [Manual]. <https://CRAN.R-project.org/package=stringr>

Wickham, H. (2020). *forcats: Tools for working with categorical variables (factors)* [Manual]. <https://CRAN.R-project.org/package=forcats>

Wickham, H., Averick, M., Bryan, J., Chang, W., McGowan, L. D., François, R., Grolemund, G., Hayes, A., Henry, L., Hester, J., Kuhn, M., Pedersen, T. L., Miller, E., Bache, S. M., Müller, K., Ooms, J., Robinson, D., Seidel, D. P., Spinu, V., … Yutani, H. (2019). Welcome to the tidyverse. *Journal of Open Source Software*, *4*(43), 1686. <https://doi.org/10/ggddkj>

Wickham, H., & Bryan, J. (2020). *usethis: Automate package and project setup* [Manual]. <https://CRAN.R-project.org/package=usethis>

Wickham, H., François, R., Henry, L., & Müller, K. (2020). *dplyr: A grammar of data manipulation* [Manual]. <https://CRAN.R-project.org/package=dplyr>

Wickham, H., & Henry, L. (2020). *tidyr: Tidy messy data* [Manual]. <https://CRAN.R-project.org/package=tidyr>

Wickham, H., Hester, J., & Chang, W. (2020). *devtools: Tools to make developing r packages easier* [Manual]. <https://CRAN.R-project.org/package=devtools>

Wickham, H., Hester, J., & Francois, R. (2018). *readr: Read rectangular text data* [Manual]. <https://CRAN.R-project.org/package=readr>

Wickham, H., & Seidel, D. (2020). *scales: Scale functions for visualization* [Manual]. <https://CRAN.R-project.org/package=scales>

Xie, Y. (2015). *Dynamic documents with R and knitr* (2nd ed.). Chapman and Hall/CRC. <https://yihui.org/knitr/>

Xie, Y. (2016). *bookdown: Authoring books and technical documents with R markdown*. Chapman and Hall/CRC. <https://github.com/rstudio/bookdown>

Xie, Y., Allaire, J. J., & Grolemund, G. (2018). *R markdown: The definitive guide*. Chapman and Hall/CRC. <https://bookdown.org/yihui/rmarkdown>

Zeileis, A., & Croissant, Y. (2010). Extended model formulas in R: Multiple parts and multiple responses. *Journal of Statistical Software*, *34*(1), 1–13. <https://doi.org/10/gd3vrb>

## Predictiveness Score Equations and Simulated Score using *ρ* ≤ 0.00

The predictiveness score is a weighted version of Type I error^[[1]](#footnote-2)^, also called alpha inflation or the familywise error rate. It is the probability of incorrectly rejecting the null hypothesis, when the null is true (i.e., at least one false positive). The formula for Type I error is as follows (e.g., Cohen et al. 2003):

$1-\left( 1-\alpha\right)^{k}=$ Type I error rate (probability)

When the null is false (here, *ρ* > 0 for one-tailed), instead of Type I error the formula can be used to calculate the probability of detecting at least one effect reaching a desired level of significance. Thus δ (statistical power) is substituted for $\alpha$ (the alpha-level for statistical significance). A priori statistical power is calculated by specifying a sample size, effect size, and *p*-value for statistical significance and then used in this equation:

$1-\left( 1-\delta\right)^{k}=$ Probability of obtaining at least one result (reaching a specified significance level)

Using the example from the paper of *N* = 24, *ρ* = 0.29, and *p <* 0.05 (one-tailed; the exact value used for calculations here is *p* $=0.04\bar{9}$), the calculated statistical power is $\delta_{sig}=41.02\%$. For *k* = 3 with the parameter values in the previous sentence, the probability of obtaining at least one significant result is:

$1-\left( 1-41.02\% \right)^{3}=79.48\%$ ($\mathrm{Probability}_{sig})$

The weight for significance results is +1, thus the expected predictiveness score is 79.48% without including marginal significance; see the last equation.

For marginal significance, *p* < 0.10 (exact value for calculations is $p=0.09\bar{9})$, power is slightly higher: $\delta_{marsig}$= $55.60\%$ (both the red and the pink areas in Figure 4 of the paper.)

$1-\left( 1-55.60\% \right)^{3}=91.25\%$ ($\mathrm{Probability}_{marsig and sig})$

Note the calculation of power for marginal significance also includes statistical significance, thus we need to calculate probability for results that only reach marginal significance (i.e., *p* < 0.10 and *p* > 0.05):

$\mathrm{Probability}_{marsig and sig}-\mathrm{Probability}_{sig}$= $\mathrm{Probability}_{marsig only}$

Continuing the example, the probability of results that are *only* marginally significant is:

$91.25\%-79.48\%=$ 11.77%

That is, there is a 12% probability of obtaining a result that is only marginally significant (the pink shaded area in Figure 4 of the paper). Note only marginally significant results have a +0.5 weight.

${(+1 \times Probability}_{sig}) \times(+0.5 {\times Probabiltiy}_{marsig only})=$ Expected Predictiveness Score

For this example, the predictiveness score is:

$(1 \times79.48\%)+$(0.5 $\times11.75\%)=$85.36%

Thus, for a paper with *N* = 24, *ρ* = 0.29, *p <* 0.05 (one-tailed), and *k* = 3, the 85% expected predictiveness score approaches a perfect score of 100%. Returning to the bottom panel of Figure 2 in the paper, this is the predictiveness score shown at *k* = 3. Note marginal significance adds negligible statistical power because it encompasses only a narrow range of effects, thus it adds just a small amount to the predictiveness score (in the paper, see the minute differences between the pink and red lines in the top panels of Figures 2 and 3 in the paper and the narrow of range of effects for marginal significance in the pink area of Figure 4).

### Simulated Predictiveness Score for *ρ* ≤ 0.00

The concerning aspects of the relationship between number of effects and predictiveness score are perhaps made clearer by assuming a true effect size less than or equal to 0 (see Figure 3). Even if a directional null hypothesis is true, the probability of finding one significant (and especially one marginal) effect only increases with more effects. This is the same concept as the familiar familywise error rate, but is perhaps not as easily recognized when couched in terms of the predictiveness score. The expected value for the predictiveness score also approaches 100% as *k* increases, again even for *ρ* ≤ 0.00. To reiterate, we do not believe the true effect size is less than or equal to zero and instead use this simulation to show the score will always increase regardless of the specified effect size. Thus, the score has no statistical value.

**Figure 3**
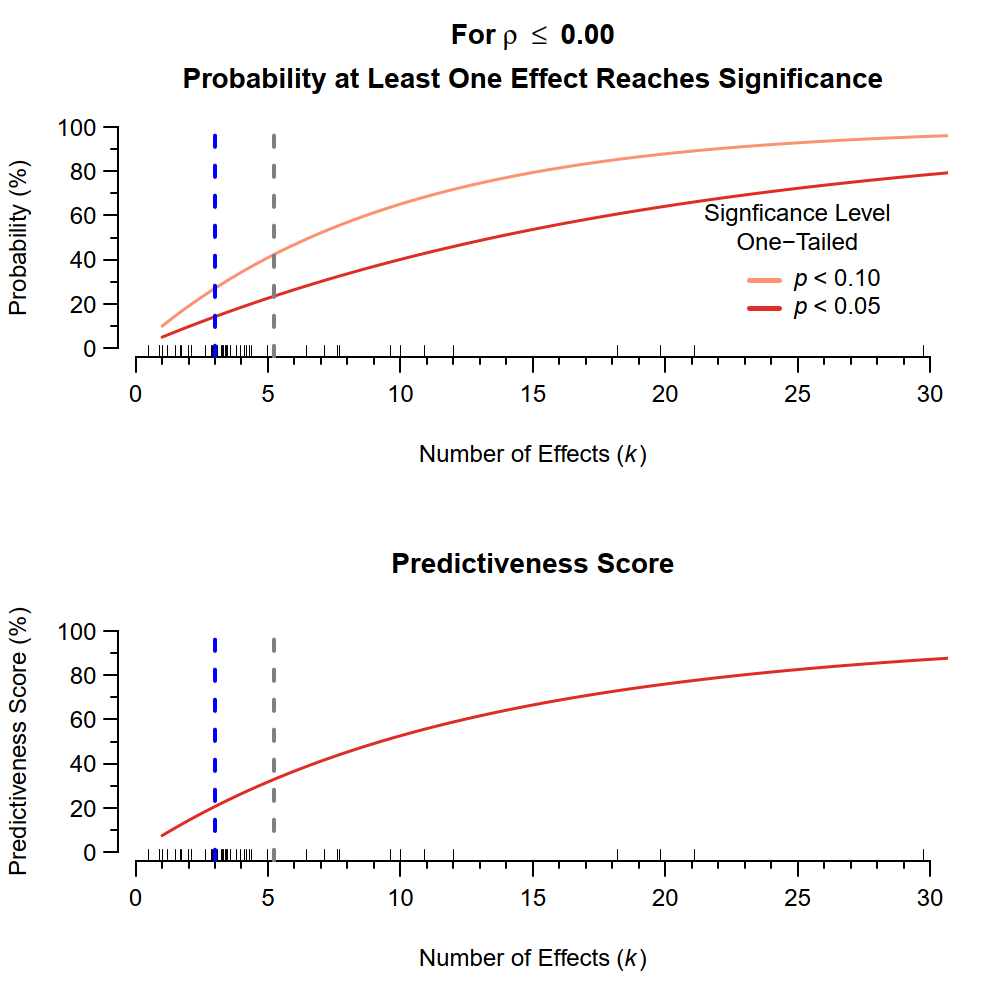
Top: The probability that at least one effect in a given paper is significant or marginally significant as a function of the number of effects per paper (*k*), defined by $1-\left( 1-\alpha\right)^{k}$, where α is determined using the significance level (α = 0.05, dark red, or α = 0.10, pink) for a true null effect size *ρ* ≤ 0.00. The median (blue) and mean (gray) number of effects per paper are shown as dotted lines. Individual *k* values for the papers in our data set are indicated by ticks above the x-axis. Bottom: The expected value of the predictiveness score as a function of *k* using an effect size *ρ* ≤ 0.00*.*

## Histograms of the Effects Below/Above Meaningful Thresholds

Beyond the comparison of means for filtered and as-reported effects, it is informative to examine the distribution of reported effects. In the context of meta-analysis, mean values summarize the distribution of effects, but can be misleading if effects are distributed non-normally and/or under high heterogeneity (Mathur and VanderWeele, 2019, 2020). For estimating the proportion of effects, we used two sets of thresholds:

1. Filtered means (approximately in the large range, *r* = 0.50; see Figure 5 and Table 1 in the paper). Note this analysis is directly relevant to Endsley.
2. Meta-analytic model means (approximately in the medium range, *r* = 0.30; see Figure 5 and Table 1 in the paper). Note this analysis is not directly relevant Endsley. We use it here as a baseline to quantify the distribution of effects relative to meta-analytic means. For a perfect unbiased estimate of the mean with normally distributed data, 50% of effects would be below the mean and 50% of effects above it. Visualization is important because it is still possible to find a 50/50 proportion, or approximately close, even with non-normally distributed effects (e.g., a bimodal or uniform distribution).

The distributions of all effects are shown in Figure 4 below using the two previously described thresholds.

**Figure 4**


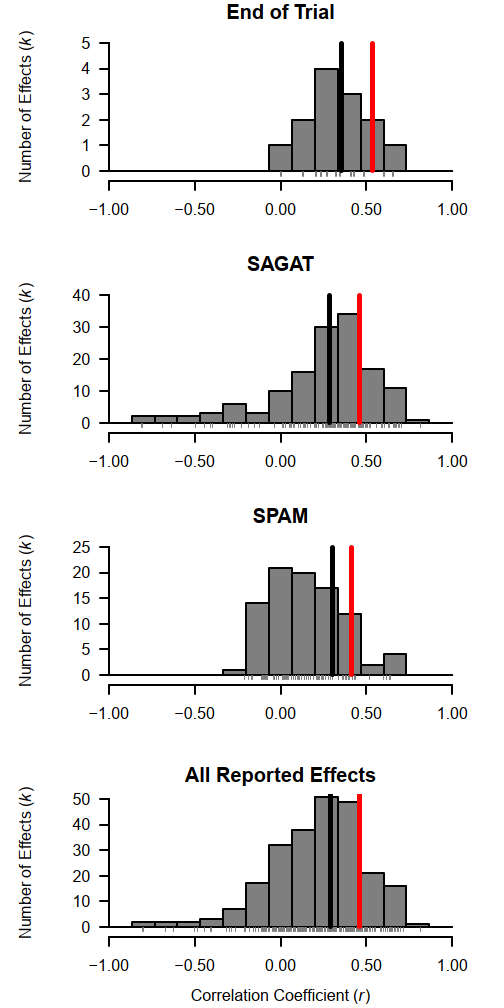


Histogram of the effects as-reported for each SA measure and across all measures for our dataset of 38 papers. Individual effect sizes are shown as ticks along the x-axis. The vertical red line depicts the mean of significance filtered effect sizes from Endsley (2019; see Table 1 in the paper) and the vertical black lines show the meta-analytic mean values (see Figure 5 in the paper). The y-axis is the number of effects in each histogram bar.

To estimate the proportions, we used the *Z*-transformed values for correlations and Fisher’s *Z* for the corresponding variances. To estimate uncertainty in proportions, confidence intervals were calculated using bootstrapping clustered by paper, and random resampling with replacement of effects within each paper. Generally, bootstrapping calculates parameter estimates by repeatedly randomly resampling independent values with replacement (Efron and Tibshirani, 1994). Here, because the effects were non-independent^[[2]](#footnote-3)^, we performed cluster bootstrapping using multiple steps^[[3]](#footnote-4)^.

Figure 5 below shows the results of these calculations. For each SA measure, the proportion of effects falling above/below the corresponding meta-analytic mean is shown in light gray and dark gray, respectively. The proportion of effects falling above and below the relevant filtered mean is shown in pink and red, respectively. As noted above, these proportions would be at 50% for perfectly representative or ideal means.

**Figure 5**


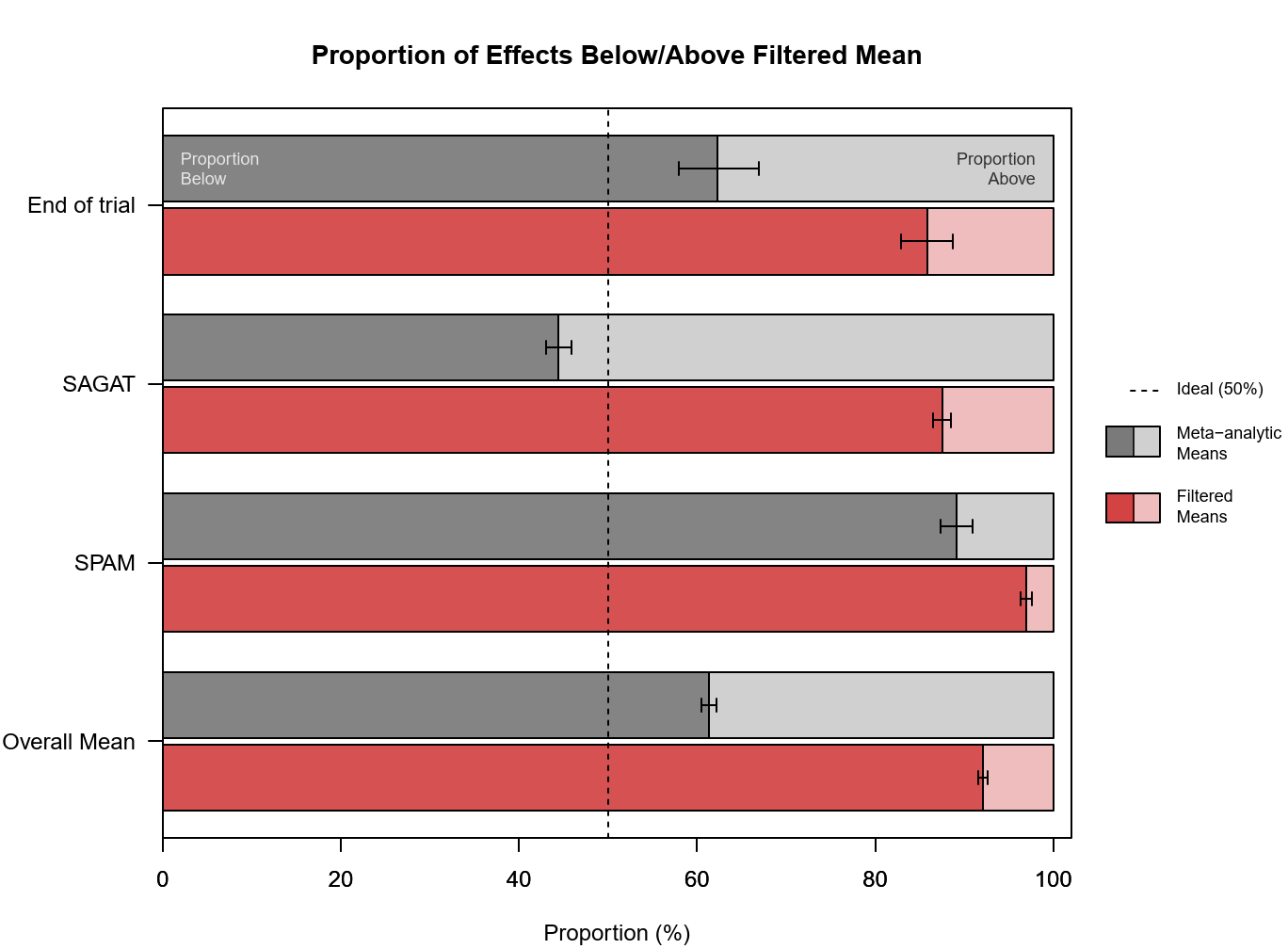


The x-axis is the proportion of all effects below specified meaningful thresholds (i.e., the point estimated effect sizes for the meta-analytic means and filtered means). Proportions of effects above and below these thresholds/means are represented by bars. Error bars represent the cluster bootstrapped 95% confidence intervals. The dashed line is the ideal proportion for a mean value.

For the filtered mean thresholds, by SA measure, more than 85% of all effects were below their threshold which indicates that selection using significance produces strongly upwardly biased estimates of effects. Although not directly relevant to Endsley (2019), three out of four SA measures were slightly to severely overestimated for thresholds using their meta-analytic means. Whereas the meta-analytic mean for the SAGAT measure was a slight underestimate. In other words, effects also tended have some concentration below their meta-analytic means (61% or 173 out of 282 effects were below the overall meta-analytic mean).

Note the filtered mean thresholds always had much higher overestimation (greater proportion of effects below) than thresholds using meta-analytic means. None of the confidence intervals overlapped. Currently, there is no inferential statistical test for comparing proportions. Confidence intervals can be visually interpreted using a rule of thumb, subject to multiple assumptions: if they barely overlap then the two-tailed *p*-value is approximately 0.01 (Cumming and Finch, 2005).

## Reproducibility Issues

A secondary problem to significance filtering was the multiple issues with reproducing statistical results from Endsley (2019). Note there are many definitions for reproducible research (Goodman et al., 2016); in this section we focus on internal statistical or computational reproducibility using data from Appendix C in Endsley in attempt to reproduce results reported in Table 5 of that paper.

To evaluate internal reproducibility, we used Endsley’s Appendix C data as-reported to reproduce means and confidence intervals for SA measures for Table 5 in Endsley, p. 13. We could only reproduce the exact value of a single SA measure: End of trial. Endsley describes using Fisher’s Z for means and with 95% confidence intervals (it is unclear how the confidence intervals were calculated). We followed the method as described in Endsley: *z*-transform the correlations, take the mean of the *z*-values, and transform the mean *z*-value back to an *r*-value. Our results are reported in Table 2. As a reminder, the original values from Endsley are reported to three decimal places and italicized.

**Table 2.** Results for mean correlations as-reported in Table 5, Endsley (2019), p**.** 13 and attempt to reproduce these results using Endsley, Appendix C data.

| Method (SA measure) |  | Mean Pearson’s *r*, Table 5 | Mean Pearson’s *r*, attempt to reproduce Table 5 using Appendix C data |
| --- | --- | --- | --- |
| End of trial |  | *0.533* | 0.533 |
| SAGAT |  | *0.459* | 0.461 |
| SPAM |  | *0.411* | 0.430 |
| Real time probes |  | *---* | 0.000 |
| SALSA |  | *0.184* | 0.202 |

We were also unable to reproduce the confidence intervals, see Table 3 below. The confidence intervals were calculated using the variance of the Fisher *Z* approximation to calculate upper and lower bounds, averaging each and then transforming them back to *r*-values. Note the miniscule range for confidence intervals reported in Endsley (2019) whereas our attempt to reproduce the confidence intervals yielded much wider values. The discrepancy in confidence intervals is enormous; it cannot be attributed to rounding errors, slight differences in means (Table 1 above), or use of the Fisher *Z* approximation for variance versus an exact calculation.

**Table 3.** Results for 95% confidence intervals correlations as-reported in Endsley (2019), Table 5, p**.** 13 and attempt to reproduce these results using Endsley, Appendix C.

| Method (SA measure) | 95% confidence interval, Table 5 | 95% confidence interval, attempt to reproduce Table 5 using Appendix C data |
| --- | --- | --- |
| End of trial | *[0.522, 0.545]* | [0.310, 0.686] |
| SAGAT | *[0.432, 0.487]* | [0.077, 0.696] |
| SPAM | *[0.368, 0.454]* | [-0.018, 0.722] |
| Real time probes | *---* | [-0.630, 0.630] |
| SALSA | *---* | [-0.586, 0.794] |

### Unweighted Mean Effect Sizes

In addition to significance filtering and issues with reproducibility, another issue for how Endsley (2019) calculated the mean effects and confidence intervals is that it appears they were unweighted means (no statement was made about weighting). Thus, a paper that contributes one effect would have the equal weight to a different paper that contributes, say, five effects or twenty effects. There are multiple methods for calculating weighted averages of effects for data with multiple and repeated measures (Borenstein et al., 2009; Scammacca et al., 2014). Weighted means are a simple alternative to fitting a multilevel meta-analytic model, but not without tradeoffs (Scammacca et al., 2014).

## Absence of Defined Inclusion and Exclusion criteria

In addition to the primary issue of significance filtering, another issue for both reproducibility and scientific quality is that Endsley (2019) lacks a critical component of meta-analysis: A systematic review with a defined and clear set of inclusion and exclusion criteria for papers and findings to be analyzed. Readers are only told that “Papers that collected experimental data with one of these techniques (or variants of them), with sufficient data reported, were included in this review” (Endsley, p. 5). However, there are no details given about what constitutes “sufficient data.” Without a (pre-)defined set of inclusion/exclusion criteria, the reader does not actually know what “sufficient data” means in this case. If there existed 20 published papers that only reported a significant positive relationship between an experimental variable and SA, but without presenting the actual data results from inferential statistical tests, one might consider such findings “sufficient data” for a quasi-quantitative vote-counting review. For an actual quantitative synthesis (i.e., a meta-analysis), however, such reports would not constitute “sufficient data.” This precludes methodological replication. Adding to the ambiguity, Endsley (2019) is an atypical hybrid analysis that employs both quasi-quantitative vote-counting and using significance to select and filter out results for quantitative synthesis.

We also identified inconsistencies in Endsley (2019) which may be due to the lack of defined criteria for inclusion/exclusion of papers and results. We provide multiple examples, with the caveat this is not an exhaustive evaluation. For example, Appendix C in Endsley contains 11 comparisons (SA measures by paper), 10 papers, with predictiveness scores but no corresponding mean effect (denoted by “---”). For most of these comparisons the reasons for inclusion/exclusion were unknown^[[4]](#footnote-5)^. In addition, two papers were given mean correlations of *r* = *0.000* for unknown reasons (we included these in calculations for Table 1 in the paper, using Appendix C values exactly as-reported). Moreover, some results were only partially included from papers with no explanation. For example, in the Loft et al. (2015) paper the SPAM measure was included but not the SAGAT measure. Another example is results in Gutzwiller and Clegg (2013): Endsley’s mean calculation uses only a single effect (the largest effect size reported), yet the paper reports a total of 10 SA-performance correlations; nine of these reported effects were significant or marginally significant.

Finally, numerous quasi-quantitative comparisons and interpretations are made throughout Endsley (2019). For example:

“Six studies included a direct comparison of the sensitivity of SAGAT and either SPAM or real-time queries in the same study. Two studies found SAGAT more sensitive than real-time probes (Endsley, Sollenberger, & Stein, 2000; Jones & Endsley, 2004), three studies found SPAM more sensitive than SAGAT…” (Endsley, p. 10).

“Two studies found SAGAT to be more predictive than real-time probes or SPAM (Jones & Endsley, 2004; Loft, Bowden, et al., 2015). Two studies found SPAM to be more predictive than SAGAT (Durso et al., 2006; Kraemer & Süß, 2015).” (Endsley, p. 11).

Given the absence of clear systematic review criteria, it is unknown if these are the only pertinent papers and results for such comparisons. Furthermore, a meta-analytic approach would synthesize and compare effect sizes including uncertainty, rather than interpreting results based on counts of statistical significance or lack thereof. Yet another issue with this quasi-quantitative approach of vote-counting, which ignores uncertainty, is that significant and non-significant results may not actually be significantly different (Gelman & Stern, 2006).

## Supplemental Material References (not cited in the manuscript)

Cumming, G., and Finch, S. (2005). Inference by eye: Confidence intervals and how to read pictures of data. *American Psychologist* 60, 170–180.

Efron, B. (1987). Better Bootstrap Confidence Intervals. *Journal of the American Statistical Association* 82, 171–185. doi:10.2307/2289144.

Efron, B., and Tibshirani, R. J. (1994). *An Introduction to the Bootstrap*. New York: CRC Press.

Field, C. A., and Welsh, A. H. (2007). Bootstrapping clustered data. *Journal of the Royal Statistical Society: Series B (Statistical Methodology)* 69, 369–390. doi:10.1111/j.1467-9868.2007.00593.x.

Gelman, A., & Stern, H. (2006). The difference between “significant” and “not significant” is not itself statistically significant. *The American Statistician*, *60*(4), 328–331. https://doi.org/10.1198/000313006X152649

Goodman, S. N., Fanelli, D., & Ioannidis, J. P. A. (2016). What does research reproducibility mean? *Science Translational Medicine*, *8*(341), 341ps12-341ps12. https://doi.org/10/gc5sjs

Hackman, J. R. (2003). Learning more by crossing levels: Evidence from airplanes, hospitals, and orchestras. *Journal of Organizational Behavior*, *24*(8), 905–922. https://doi.org/10.1002/job.226

Storey, J. D., & Tibshirani, R. (2003). Statistical significance for genomewide studies. *Proceedings of the National Academy of Sciences*, *100*(16), 9440–9445. https://doi.org/10.1073/pnas.1530509100

1. Type I error is related to the False Discover Rate (FDR). The distinction is the FDR is the total percentage of significant results that are true null results (Storey & Tibshirani, 2003). In contrast, Type I error is the rate for at-least-one false positive (not the total rate of all false positives) given the null hypothesis is true. [↑](#footnote-ref-2)
2. Conceptual explanation of cluster bootstrapping: The values, here effect sizes, can be represented by different colored marbles in a single large bag. However, rather than there being a single bag, most papers/studies (separate bags) contain multiple effects thus the effects (marbles) are clustered within different papers. To address this, we used a cluster or block bootstrap (Field and Welsh, 2007) by paper. Extending the marble example, this can be conceptualized by clustering using separate bags (papers or multiple studies within the same paper) each containing their respective varying distributions of marbles with different distributed (effects). The marbles in each bag are then each randomly sampled a number of times. Next, the separately sampled bags are combined to create a set of bootstrap samples; representative of clustering. Last, the bootstrap samples are used to estimate the sampling mean and confidence intervals for different colored marbles for all bags. Note for estimating bootstrapped means (of effects), clustering versus independent resampling produce equivalent values. Whereas, the confidence intervals for each estimation method differ and are wrong when independence is incorrectly assumed instead of using cluster bootstrapping. [↑](#footnote-ref-3)
3. Implementation of cluster bootstrapping, steps: First, we created 50 bootstrapped samples clustered by paper (for each SA measure). Second, for each bootstrapped sample, we estimated the proportion of effects (Mathur and VanderWeele, 2019, 2020) below the filtered and meta-analytic thresholds described above. Proportions (below) are denoted by $\hat{p}.$ Third, we used the 50 $\hat{p}$values to calculate the average proportion of effects, below the specified threshold, with confidence intervals calculated using the bias-corrected and accelerated bootstrapping procedure (Efron, 1987). [↑](#footnote-ref-4)
4. There are potential reasons for omitting the mean effects in four papers. One paper had a predictiveness score of zero, thus it might have been excluded by filtering. Two other papers reported a Spearman correlation instead of a Pearson correlation. A Spearman correlation (or point biserial correlation) is a non-parametric version of a Pearson correlation for rank order data; both tests often produce similar results (Cohen et al., 2003). Ideally, the Spearman correlation results should have been included because no conversion was required. Last, one paper only reported an approximate *p*-value with no effect size, which we also excluded here (see Supplemental Material 1.1). [↑](#footnote-ref-5)
